# Histone bivalency regulates the timing of cerebellar granule cell development

**DOI:** 10.1101/gad.350594.123

**Published:** 2023-07-01

**Authors:** Kärt Mätlik, Eve-Ellen Govek, Matthew R. Paul, C. David Allis, Mary E. Hatten

**Affiliations:** 1 Laboratory of Developmental Neurobiology, Rockefeller University, New York, New York 10065, USA;; 2 Bioinformatics Resource Center, Rockefeller University, New York, New York 10065, USA;; 3 Laboratory of Chromatin Biology and Epigenetics, Rockefeller University, New York, New York 10065, USA

**Keywords:** H3K27me3, cerebellum, glial-guided migration, granule cells, histone bivalency, mouse

## Abstract

Developing neurons undergo a progression of morphological and gene expression changes as they transition from neuronal progenitors to mature neurons. Here we used RNA-seq and H3K4me3 and H3K27me3 ChIP-seq to analyze how chromatin modifications control gene expression in a specific type of CNS neuron: the mouse cerebellar granule cell (GC). We found that in proliferating GC progenitors (GCPs), H3K4me3/H3K27me3 bivalency is common at neuronal genes and undergoes dynamic changes that correlate with gene expression during migration and circuit formation. Expressing a fluorescent sensor for bivalent domains revealed subnuclear bivalent foci in proliferating GCPs. Inhibiting H3K27 methyltransferases EZH1 and EZH2 in vitro and in organotypic cerebellar slices dramatically altered the expression of bivalent genes, induced the down-regulation of migration-related genes and up-regulation of synaptic genes, inhibited glial-guided migration, and accelerated terminal differentiation. Thus, histone bivalency is required to regulate the timing of the progression from progenitor cells to mature neurons.

## Abstract (precis)

In this study, Mätlik et al. used RNA-seq and H3K4me3 and H3K27me3 ChIP-seq to analyze how chromatin modifications control gene expression in progenitor cerebellar granule cells. Inhibition of H3K27 methyltransferases EZH1/2 perturbed glial-guided migration while accelerating neuronal maturation, and this study demonstrates that temporally regulated H3K4me3/H3K27me3 bivalency influences neurodevelopmental gene expression, lineage specification, migration, and differentiation.

Neuronal development is controlled by gene expression programs that regulate the developmental progression of neuronal progenitor cells as they undergo glial-guided migration and circuit formation. These developmental gene expression programs are thought to be controlled by epigenetic mechanisms, including changes in chromatin accessibility and modifications of histones and DNA (Strahl and Allis 2000; Jenuwein and Allis 2001; Dixon et al. 2015). However, even though epigenetic dysregulation is increasingly recognized as an important contributor to neurodevelopmental diseases (Serrano 2018), the precise mechanisms by which changes in chromatin control gene expression during neurogenesis, glial-guided migration, and maturation in specific types of CNS neurons are largely unknown.

The cerebellar cortex has long provided an important model system for neuronal development, and studies on the cerebellar granule cell (GC) have identified molecular mechanisms that control glial-guided migration (Edmondson and Hatten 1987; Solecki et al. 2004, 2009; Horn et al. 2018) and circuit formation (Brickley et al. 1996; Watt et al. 2009; Zhu et al. 2016; van der Heijden and Sillitoe 2021). Developing cerebellar GCs are an excellent model for addressing the role of chromatin in neuronal development because they are an extremely numerous yet relatively homogenous population of neuronal progenitors that undergo stereotypic and well-characterized developmental steps to become fully functional CNS neurons. GCs are derived from a pool of committed GC progenitors (GCPs) that undergo prolonged clonal expansion in the external granule layer (EGL) of the developing cerebellar cortex (Espinosa and Luo 2008; Consalez et al. 2021). Following cell cycle exit, immature GCs extend bipolar neurites (parallel fibers) and form a leading process along the radial glia fibers to undergo glial-guided migration. Postmigratory GCs settle in the internal granule layer (IGL) and become multipolar as they extend dendrites and form synapses with ingrowing mossy fiber afferents to form the cerebellar circuitry.

We recently discovered that dramatic changes in the expression of chromatin-modifying genes, including DNA and histone methyltransferases, occur in GCs during the formation of the cerebellar circuitry (Zhu et al. 2016). This finding suggests that regulation of chromatin landscape is critical for GC development. One family of genes that changed significantly encodes the TET enzymes, which control the formation of 5-hydroxymethylcytosine, a DNA modification that we found is required for the transition from migratory to postmigratory GCs through the regulation of ion channel and axon guidance genes (Zhu et al. 2016). These studies from our laboratory and others (Stoyanova et al. 2021) have therefore begun to reveal how changes in DNA modifications impact neuronal development.

Histone post-translational modifications (PTMs) are chemical changes to histones that modulate DNA packaging and chromatin accessibility to transcription factors. Importantly, although histone methylation represents one of the most diverse classes of epigenetic modifications, the roles of individual histone PTMs and their combinations during the development of specific neuron types in vivo are only beginning to be understood. Two crucial histone PTMs associated with gene expression are trimethylation of histone 3 at lysine 4 (H3K4me3) and lysine 27 (H3K27me3). H3K4me3 is localized at gene promoters and generally correlates with active gene expression (Santos-Rosa et al. 2002; Bernstein et al. 2005). The regulation of H3K4me3 is critical for development, as loss of function in the methyltransferase genes that generate this modification results in embryonic lethality (Cenik and Shilatifard 2021). In contrast, H3K27me3 is a repressive modification that is enriched on developmentally silenced genes (Barski et al. 2007; Mikkelsen et al. 2007). During embryonic stem cell development, H3K27me3 is thought to repress the expression of selected genes in a lineage-specific manner and thus contribute to lineage decisions during differentiation (Mohn et al. 2008). Loss of H3K27 methyltransferase EZH2 or other components of the Polycomb repressive complex 2 (PRC2) during early neural development induces profound transcriptional dysregulation and impaired neuronal progenitor proliferation, neuronal cell type specification, and differentiation (Hirabayashi et al. 2009; Zhang et al. 2014; Feng et al. 2016), demonstrating that regulation of H3K27me3 is essential at early stages of development.

At most genomic loci, H3K4me3 and H3K27me3 are mutually exclusive, and maintaining their balance is critical for gene expression regulation during development (Piunti and Shilatifard 2016; Cenik and Shilatifard 2021). However, during lineage commitment and cell fate determination in pluripotent cells, these two modifications often colocalize at the promoters of developmentally regulated genes (Bernstein et al. 2006; Mikkelsen et al. 2007; Mohn et al. 2008). These so-called bivalent domains are proposed to poise key developmental genes for subsequent lineage-specific activation (Bernstein et al. 2006) or to protect reversibly repressed genes from irreversible silencing (Kumar et al. 2021). Studies in ESC cultures have shown that during developmental progression from ESCs to neuronal progenitor cells, most bivalent promoters reduce to a monovalent status by retaining just one of the two modifications (Mikkelsen et al. 2007). The role of bivalency beyond the regulation of lineage decisions in pluripotent cells is still under investigation, and the question remains as to whether bivalent promoters are present in committed cells, including CNS neuronal progenitors, and, if so, whether they are remodeled during neuronal differentiation and maturation. It also remains to be determined how the regulation of H3K27me3 at bivalent and H3K27me3-only genes controls gene expression and developmental progression in immature neurons.

Here, we investigated how the histone modifications H3K4me3 and H3K27me3 regulate glial-guided migration and maturation of a specific type of CNS neuron: the cerebellar GC. We identified H3K4me3/H3K27me3 bivalent domains in proliferating GC progenitors and show that they are enriched at the promoters of neuronal genes. Bivalent domains are dynamically remodeled during GC development, primarily through the regulation of H3K27me3, correlating with developmental gene expression changes. Last, we found that perturbing bivalency through the inhibition of EZH1 and EZH2 inhibited glial-guided migration and accelerated neuronal maturation. Together, these data implicate histone bivalency as a specific epigenetic feature controlling the timing of gene expression during cerebellar cortex development and circuit formation.

## Results

### Dynamics of histone PTMs during cerebellar GC development

**Figure 1.**
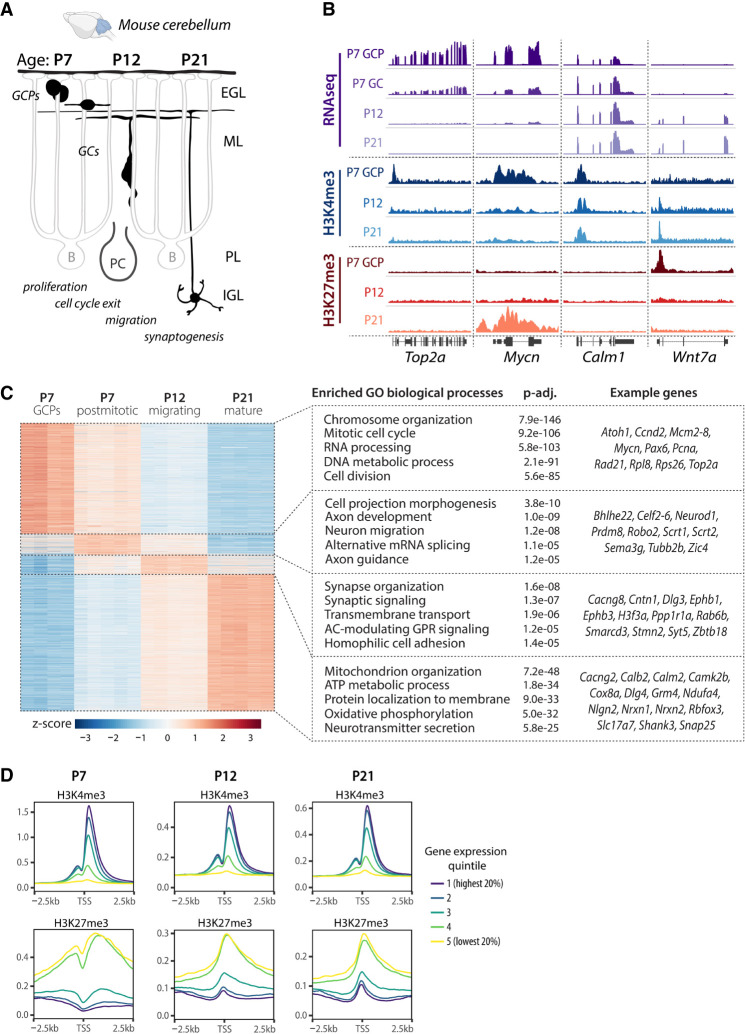
Characterization of gene expression and chromatin landscape in developing mouse cerebellar granule cells. (*A*) Schematic of postnatal GC development. During late embryogenesis and the first postnatal week, granule cell progenitors (GCPs) undergo clonal expansion in the external granule cell layer (EGL). Upon exiting the cell cycle, immature GCs extend parallel fibers and a leading process and begin glial-guided migration along Bergmann glia fibers. After passing the Purkinje cell layer (PL), the GCs stop migrating and extend dendrites, forming synapses with mossy fiber afferents to form the cerebellar circuitry. (IGL) Internal granule layer, (ML) molecular layer, (P) postnatal day. (*B*) Genome browser view of representative genes undergoing gene expression and histone modification changes during GC development. Representative ChIP-seq and TRAP RNA-seq samples are shown. H3K4me3 (*n* = 2–3 samples/group) and H3K27me3 (*n* = 4–5 samples/group) ChIP-seq was performed on chromatin isolated from GCPs (P7) or from GC nuclei sorted using FANS (P12 and P21). RNA-seq was performed on TRAP RNA isolated from P5–P7 *Tg(Atoh1-Egfp-L10a)* GCPs (P7-GCP; *n* = 4 samples/group) or from the cerebellar lysates of *Tg(Neurod1-Egfp-L10a)* mice at P7 (P7-GC), P12, and P21 (*n* = 5 mice/group). (*C*, *left*) Heat map depicting differentially expressed (DE) genes in developing GCs. DE genes (*P-*adj < 0.01, log_2_FC ≥ 1) were identified by pairwise comparisons between groups using DESeq2 and sorted by the highest expressed genes at each age. (*Right*) Gene ontology (GO) analysis of the highest expressed genes at each developmental time point, identified using clusterProfiler. The GO biological process categories were sorted by the adjusted *P*-value, and the top five enriched nonredundant categories are shown for each age. (*D*) Relative signal of the histone modifications H3K4me3 and H3K27me3 around transcription start sites (TSSs) at P7, P12, and P21, grouped into quintiles based on gene expression levels.

The genomic localization of histone PTMs is well established in a range of pluripotent and differentiated cells. However, the dynamics of histone PTMs during the development of specific types of CNS neurons in vivo are less well studied. To understand how histone PTMs regulate gene expression during the development of a specific type of neuron, we isolated chromatin and RNA from mouse cerebellar GCs at postnatal day 7 (P7), P12, and P21. These ages correspond to GC progenitor proliferation, glial-guided migration, and neuronal maturation, respectively (Fig. 1A). We then characterized the dynamics of histone PTMs H3K4me3 and H3K27me3, as well as gene expression at these key stages of neuronal development. To determine the genomic localization of H3K4me3 and H3K27me3 across GC development, we performed chromatin immunoprecipitation combined with sequencing (ChIP-seq) from chromatin isolated from proliferating GCPs (as described in Hatten 1985) and postmitotic GC nuclei isolated with fluorescence-activated nucleus sorting (Fig. 1B; Xu et al. 2018). To confirm that the isolation methods yielded cell populations enriched for GCs, we evaluated the levels of H3K4me3 at marker genes of different cerebellar cell types identified based on a published scRNA-seq data set from P60 mouse brains (Supplemental Table S1; Saunders et al. 2018). We found high levels of H3K4me3 at *Neurod1*, which is known to be highly expressed in GCs (Behesti et al. 2021), but little or no H3K4me3 signal at genes specific to other cerebellar cell types (Supplemental Fig. S1A). These results therefore showed that the isolation methods yielded chromatin from pure populations of GCs.

To relate histone PTM changes to changes in gene expression, we used the translating ribosome affinity purification (TRAP) (Doyle et al. 2008; Heiman et al. 2008) and performed RNA-seq on translating mRNA isolated from *Tg(Atoh1-Egfp-L10a)* and *Tg(Neurod1-Egfp-L10a)* mice to characterize gene expression in proliferating and postmitotic GCs, respectively (Supplemental Fig. S1A–C). At each developmental time point, known GC-specific genes were enriched in the TRAP samples compared with the input, whereas marker genes of other cerebellar cell types were depleted (Supplemental Fig. S1A,D). In addition, the expression of marker genes of other cerebellar cells was significantly lower than the expression of GC-specific genes in TRAP RNA (Supplemental Fig. S1E), demonstrating that the TRAP methodology yielded GC-enriched RNA.

We then determined the expression patterns of developmentally regulated genes in GCs at each developmental stage. To that end, we performed pairwise differential gene expression analysis using DESeq2. Genes with a log_2_FC ≥ 1 and *P-*adj < 0.01 in any pairwise comparisons were considered to be differentially expressed. For each gene, we determined the developmental stage where this gene was expressed at the highest level and subsequently performed gene ontology (GO) enrichment analysis to identify the biological processes enriched at each developmental stage (Fig. 1C; Supplemental Table S2). At P7, the enriched pathways in proliferating GCPs were associated with the cell cycle, whereas newly differentiated, postmitotic GCs predominantly expressed genes involved in axon extension and axon guidance. In migrating GCs at P12, genes expressed at the highest levels were related to neuronal differentiation and synaptic signaling, whereas pathways involved in mitochondrial function and neurotransmitter secretion reached their highest expression in mature GCs at P21. These data were consistent with a previously published microarray data set from our laboratory (Zhu et al. 2016). We next performed a genome-wide comparison of ChIP-seq and RNA-seq data and found that the levels of H3K4me3 and H3K27me3 at the promoter correlated with gene expression, with the H3K4me3 signal being highest at highly expressed genes and H3K27me3 signal being highest at repressed genes (Fig. 1D).

### GCPs exhibit developmentally regulated H3K4me3/H3K27me3 bivalency

**Figure 2.**
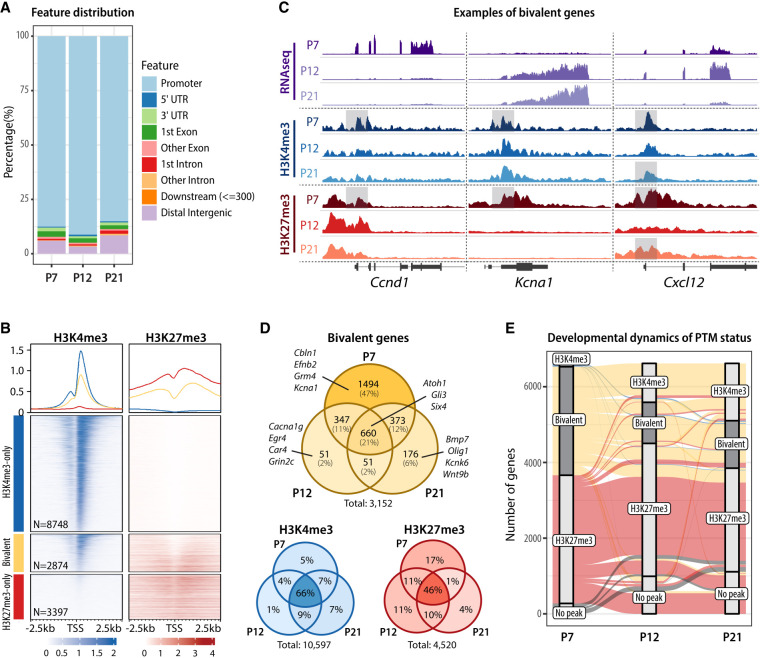
H3K4me3/H3K27me3 bivalent promoters are prevalent in proliferating GC progenitors. (*A*) Genomic distribution of bivalent peaks. Bivalent peaks were defined as regions where H3K4me3 and H3K27me3 peaks overlapped. The genomic distribution of the identified bivalent peaks was determined using the ChIPseeker package. (*B*) Enrichment map depicting ChIP-seq normalized reads in P7 GCPs, centered at TSS ± 2.5 kb, sorted by H3K4me3 signal, and grouped by TSS status (H3K4me3-only, bivalent, or H3K27me3-only). (*C*) Genome browser view of representative bivalent genes. Shaded areas denote the bivalent regions around the TSS. (*D*) Venn diagrams depicting the number of bivalent, H3K4me3-only, and H3K27me3-only genes across GC development. Examples of bivalent genes are shown. Note that bivalent genes are particularly common in P7 GCPs, while the majority of H3K4me3-only and H3K27me3-only genes are stable between P7 and P21. (*E*) Alluvial plot showing the dynamics of histone post-translational modifications between P7 and P21. Bars represent PTM statuses at promoters, and lines indicate the changes in PTMs over development. Only genes that are either bivalent or H3K27me3-only at any age are shown. Bivalent genes are highlighted with dark-gray bars. Groups that contain <0.5% of included genes were omitted for simplicity. Note that a major fraction of P7 bivalent genes become H3K4me3-only by P12 or P21, whereas most P7 H3K27me3-only genes remain H3K27me3-only until P21.

In addition to identifying genes with H3K4me3 and H3K27me3 marks at specific developmental stages, we found that these modifications often fully or partially overlapped, forming bivalent domains. Bivalent regions localized almost exclusively to promoters (Fig. 2A). We determined H3K4me3 and H3K27me3 profiles at H3K4me3-only, bivalent, and H3K27me3-only genes at P7 and found that both H3K4me3 and H3K27me3 signals were on average higher at monovalent than at bivalent loci (Fig. 2B). We noticed that genes with bivalent promoters were often bivalent in GCPs and resolved to monovalent in mature GCs (Fig. 2C). For example, genes encoding the cell cycle protein cyclin D1 (*Ccnd1*) and the voltage-gated potassium channel subunit *Kcna1* were both bivalent at P7, but during migration and maturation, *Ccnd1* retained H3K27me3 with a corresponding decrease in gene expression, while *Kcna1* retained H3K4me3 with a corresponding increase in gene expression (Fig. 2C). We also identified genes that underwent a more dynamic change in bivalency, like *Cxcl12*, for example, which encodes a chemokine involved in GC glial-guided migration (Ozawa et al. 2016). *Cxcl12* was bivalent at P7 and P21, but during glial-guided migration at P12, H3K27me3 was transiently removed, correlating with a peak in *Cxcl12* expression in migrating cells (Fig. 2C).

Across all developmental stages, we identified a total of >3000 bivalent genes (Fig. 2D; Supplemental Table S3), demonstrating that bivalency is common in this committed CNS neuron progenitor population. The number of bivalent genes was high in progenitor cells and markedly lower in migrating and mature neurons (Fig. 2D). Approximately 50% of genes bivalent at P7 resolved to H3K4me3-only by P21, demonstrating that H3K27me3 was actively removed from bivalent loci during GC differentiation and maturation (Fig. 2E). In contrast, the methylation pattern of genes that only had H3K4me3 or H3K27me3 was relatively stable as development progressed (Fig. 2D,E; Supplemental Fig. S2A). Moreover, regulation of H3K27me3 was more dynamic at bivalent loci than at H3K27me3-only loci: Most (63%) P7 H3K27me3-only peaks maintained their H3K27me3-only status throughout GC development, whereas H3K27me3 was removed from 51% of bivalent promoters by P21 (Fig. 2E). These results suggest that monovalent genes retain relatively stable methylation status, while PTMs at bivalent genes are dynamically regulated during development.

### Bivalent domains exist in individual GCPs

**Figure 3.**
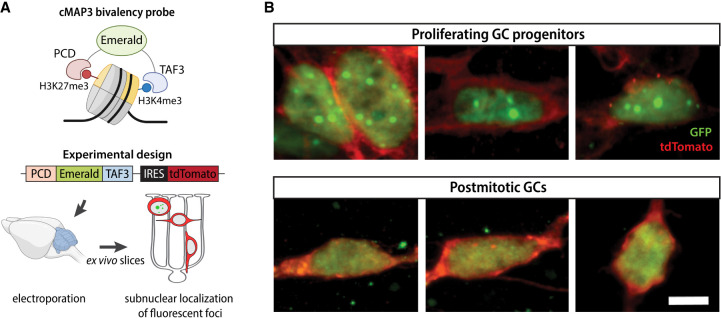
Identification of bivalent domains ex vivo. (*A*) Schematic of the cMAP3 bivalency probe used to detect truly bivalent domains ex vivo. The probe consists of a fluorophore (Emerald) fused with H3K27me3 and H3K4me3 reader domains. The plasmid coexpresses tdTomato under IRES for assessing GC morphology. (*B*) Representative images of GCs electroporated with the cMAP3 bivalency probe, showing the subnuclear localization of bivalent domains. Scale bar, 5 µm.

To address the question of whether bivalent domains exist in individual GCs or are an averaged feature of the GC population, we used a recently developed multivalent chromatin sensor (cMAP3) that selectively binds H3K4me3/H3K27me3 bivalent nucleosomes (Fig. 3A, top panel; Delachat et al. 2018; Hoffman et al. 2020). This genetically encoded sensor contains a fluorescent reporter fused to a H3K27me3-binding Polycomb chromodomain (PCD) at the N terminus and a H3K4me3-binding plant homeodomain (PHD) derived from TFIID subunit 3 (TAF3) at the C terminus. The binding of both reader domains to bivalent nucleosomes leads to the stabilization of fluorescent signal and the appearance of characteristic fluorescent foci at bivalent domains (Delachat et al. 2018; Hoffman et al. 2020). The plasmid also expresses tdTomato from an internal ribosome entry site, allowing us to simultaneously assess GC morphology.

To detect bivalent chromatin in GCs at defined stages of development, we electroporated cerebella from P8 mice with a plasmid coexpressing the cMAP3 construct and the tdTomato reporter (Fig. 3A, bottom panel), generated ex vivo organotypic slices, and imaged cMAP3-expressing cells after 6–72 h. As we previously showed, electroporation of the cerebella at this age results in specific targeting of GCPs that reside in the EGL. Importantly, GCs undergoing specific stages of development have the same morphology and laminar position in the ex vivo slices as they do in vivo (Fig. 1A). In addition, GC development in slices follows the same schedule as in vivo. Therefore, the ex vivo slice culture system allows us to selectively target GCPs and identify GCs at specific stages of development using their morphology and location in the tissue (Solecki et al. 2004; Zhu et al. 2016; Govek et al. 2018).

We imaged cMAP3-expressing GCs at various time points after electroporation. The developmental stage of proliferating GCPs and postmitotic GCs was inferred based on cell morphology and evaluated by tdTomato fluorescence. Cycling GCPs in the outer EGL exhibit a range of complex, dynamic morphologies and can have extensive, elaborate protrusions (Hanzel et al. 2019), while newly differentiated GCs in the lower EGL extend bipolar parallel fiber processes. During migration, GCs extend a leading process in the direction of migration while trailing a T-shaped axon behind the soma and become multipolar as they mature. We found that proliferating GCPs imaged 6–12 h after electroporation displayed distinct fluorescent subnuclear foci, whereas postmitotic GCs imaged 48–72 h after electroporation exhibited a diffuse fluorescence pattern without detectable subnuclear foci (Fig. 3B). These results show that individual GCPs contain bivalent domains and support our observation that bivalency is more prevalent in proliferating progenitor cells than in postmitotic cells (Fig. 2C,D).

### Bivalency is enriched at developmentally regulated neuronal genes

**Figure 4.**
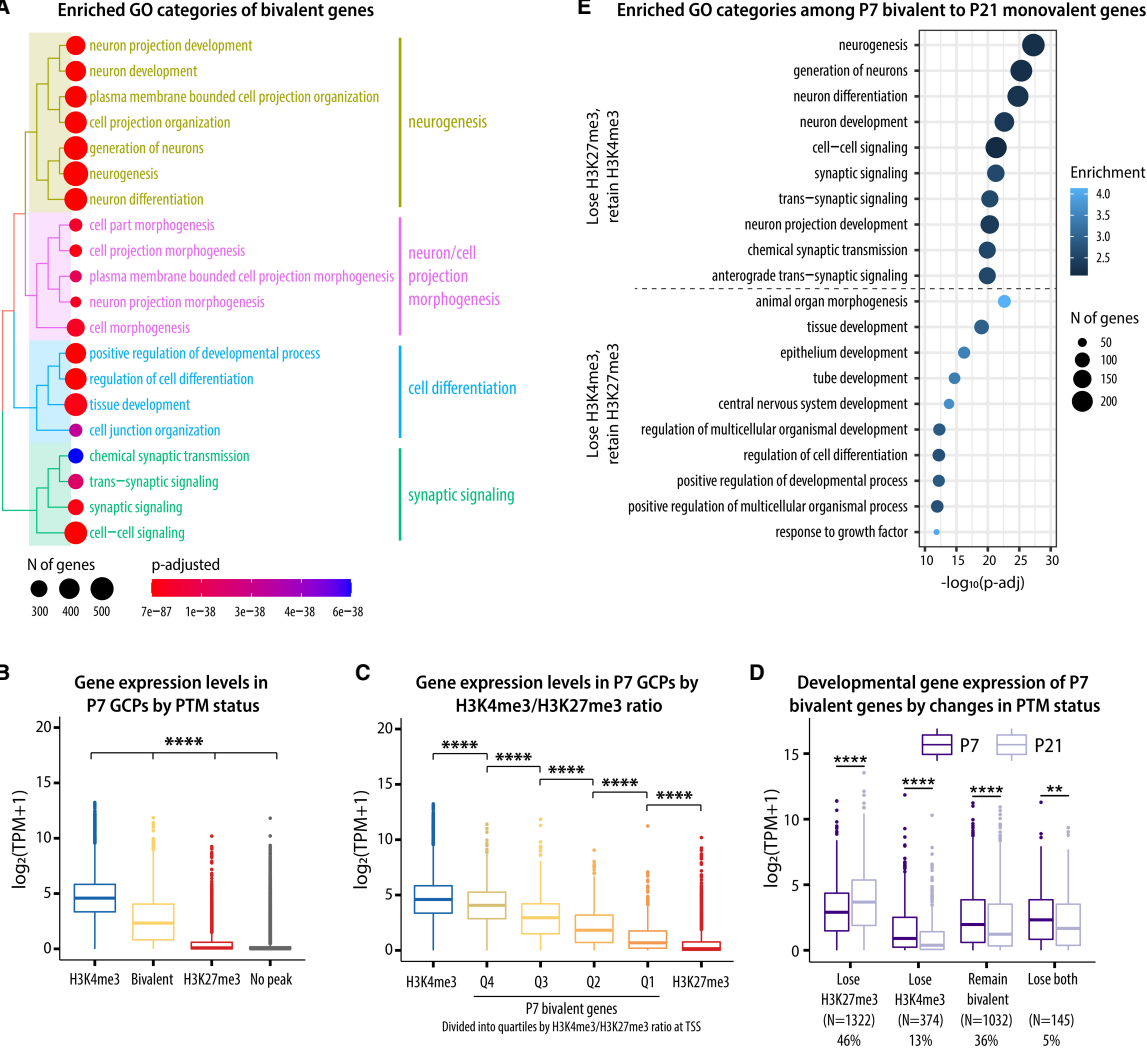
H3K4me3/H3K27me3 bivalency predicts gene expression levels in GCs. (*A*) Enriched GO (biological process) categories of bivalent genes identified using the clusterProfiler package. The top 20 identified categories were plotted using the treeplot function to visualize the relationship between the categories. (*B*) At P7, bivalent genes are expressed at lower levels than H3K4me3-only genes but at higher levels than H3K27me3 or no-peak genes. Kruskal–Wallis one-way ANOVA followed by Dunn's post hoc test. (****) *P* < 0.0001 between all comparisons. (*C*) The expression of bivalent genes correlates with H3K4me3/H3K27me3 ratio at the TSS. One-way ANOVA followed by Tukey HSD post hoc test. Correction for multiple comparisons was performed considering all comparisons, but only significances between adjacent groups are shown. (****) *P* < 0.0001. (*D*) Changes in the PTM status of bivalent genes correlate with gene expression across GC development. Pairwise *t*-test, adjusted for multiple comparisons using the BH method. (**) *P* < 0.01, (****) *P* < 0.0001. (*E*) Enriched GO (biological process) categories among genes that change from bivalent to monovalent between P7 and P21, identified with clusterProfile.

Having confirmed that bivalent domains exist in individual GCs, we asked which biological processes were enriched among bivalent genes. Toward that end, we performed gene ontology (GO) analysis of all genes that were bivalent between P7 and P21 and found that bivalency is common at genes associated with key functions during neuronal development, including neurogenesis, migration, and synaptic function (Fig. 4A; Supplemental Tables S2, S3). We also determined the enriched categories of bivalent and H3K27me3-only genes separately for each developmental stage. Interestingly, we found that the enriched categories of both bivalent and H3K27me3-only genes were remarkably stable between P7 and P21, with bivalent genes being associated with neuronal development and H3K27me3-only genes being associated with earlier developmental processes, such as organ morphogenesis and pattern specification (Supplemental Fig. S2B; Supplemental Table S2). This suggested that genes marked with both H3K4me3 and H3K27me3 are functionally distinct from genes with only H3K27me3 and further implied that the regulation of bivalency at neuronal genes could be associated with gene expression regulation during development.

Based on our observation that bivalent genes are especially enriched among neuronal genes, we next asked how developmental changes in bivalency correlate with gene expression during GC development. We analyzed the expression of genes with different PTM statuses at promoters (H3K4me3-only, bivalent, H3K27me3-only, and no peak) and found that genes with bivalent promoters were expressed at lower levels compared with genes with H3K4me3 alone but at higher levels than genes with only H3K27me3 or genes without either of the peaks (Fig. 4B; Supplemental Fig. S3A). Moreover, the ratio of H3K4me3 to H3K27me3 at bivalent genes was positively correlated with gene expression (Fig. 4C), showing that the balance between H3K4me3 and H3K27me3 signals could be relevant for the regulation of gene expression levels.

We then analyzed the relationship between histone PTM changes and gene expression during GC development and found that changes in chromatin correlated with changes in gene expression levels (Fig. 4D; Supplemental Fig. S3B). Interestingly, most genes bivalent in GCPs were differentially expressed during development (44% up-regulated and 38% down-regulated by P21) (Supplemental Fig. S3C). GO analysis revealed that the genes that lost H3K27me3 and retained H3K4me3 were involved with neuronal differentiation and maturation, whereas the genes that lost H3K4me3 and retained H3K27me3 were associated with functional categories related to earlier developmental processes, such as tissue development and morphogenesis (Fig. 4E). Therefore, these results suggested that the regulation of H3K4me3 and H3K27me3 levels at bivalent domains has the potential to control and fine-tune the expression of genes required for GC maturation and that both activation and repression of bivalent genes are required for GC maturation.

### Transcription factors that determine GC identity are bivalent in developing GCs

**Figure 5.**
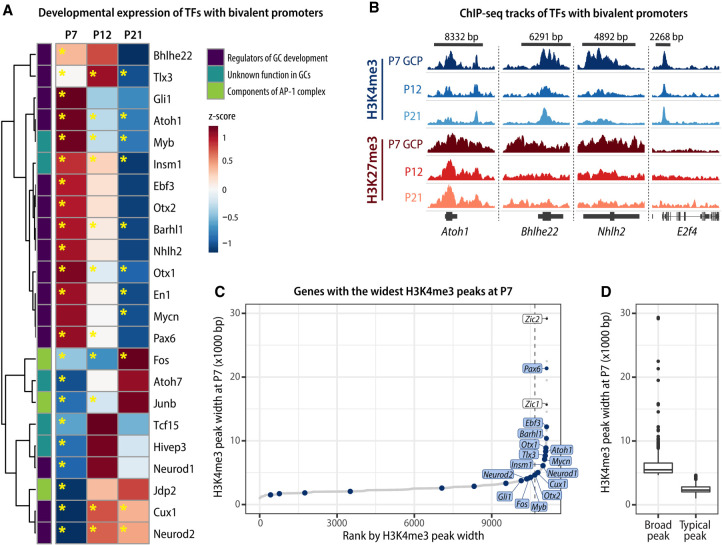
Transcription factors that establish GC identity are bivalent. (*A*) Heat map depicting the developmental gene expression dynamics of selected transcription factors with bivalent promoters. Yellow asterisks denote the time points when the genes are bivalent. (*B*) Integrative Genome Browser view of H3K4me3 and H3K27me3 signal at selected transcription factors during GC development. The indicated bivalent TFs are marked with broad domains of H3K4me3, which are found on genes involved in maintaining cellular identity. Peak widths are indicated at the *top* of the tracks. (*C*) P7 H3K4me3 peaks ranked by width in base pairs. The dashed line denotes the cutoff point between “broad” (*n* = 495) and “typical” (*n* = 10,673) peaks, determined using the elbow point method. Peaks annotated to the promoters of bivalent TF genes are shown in blue. (*D*) Widths of broad and typical H3K4me3 peaks in proliferating GCPs at P7.

After establishing that dynamics of bivalency correlated with gene expression, we asked whether bivalency could control the expression of transcription factors (TFs) that regulate GC developmental progression. We identified a total of 107 bivalent TFs with an average TPM of >5. Bivalent TFs included almost all genes known to be essential for specifying GC identity and for GC differentiation, including *Atoh1*, *Mycn*, *Pax6*, *Gli1*, and *Neurod1* (Fig. 5A). The exceptions were the *Zic* genes that were not bivalent at any age. Moreover, several TFs of the AP-1 complex that regulates activity-dependent gene expression were bivalent in developing GCs (Fig. 5A).

We noticed that many bivalent TF genes had very broad H3K4me3 peaks (Fig. 5B), which is an epigenetic signature of cell identity genes (Benayoun et al. 2014). We therefore identified all genes with the broadest H3K4me3 peaks by ranking H3K4me3 peaks by width and using the elbow point method to determine the cutoff between broad and typical peaks (Fig. 5C,D; Whyte et al. 2013). We found that H3K4me3 peaks at many bivalent TF genes were classified as broad H3K4me3 peaks, consistent with the known importance of these genes in GC development (Fig. 5A–C). Notably, the bivalent TFs with broad H3K4me3 signal included genes that were only bivalent at specific stages, as well as genes bivalent throughout GC development (Fig. 5A–C). Therefore, the TFs that specify and maintain GC identity undergo dynamic changes in their bivalency status during GC neurogenesis, glial-guided migration, and maturation.

### Perturbations in bivalency inhibit glial-guided migration and accelerate terminal differentiation

**Figure 6.**
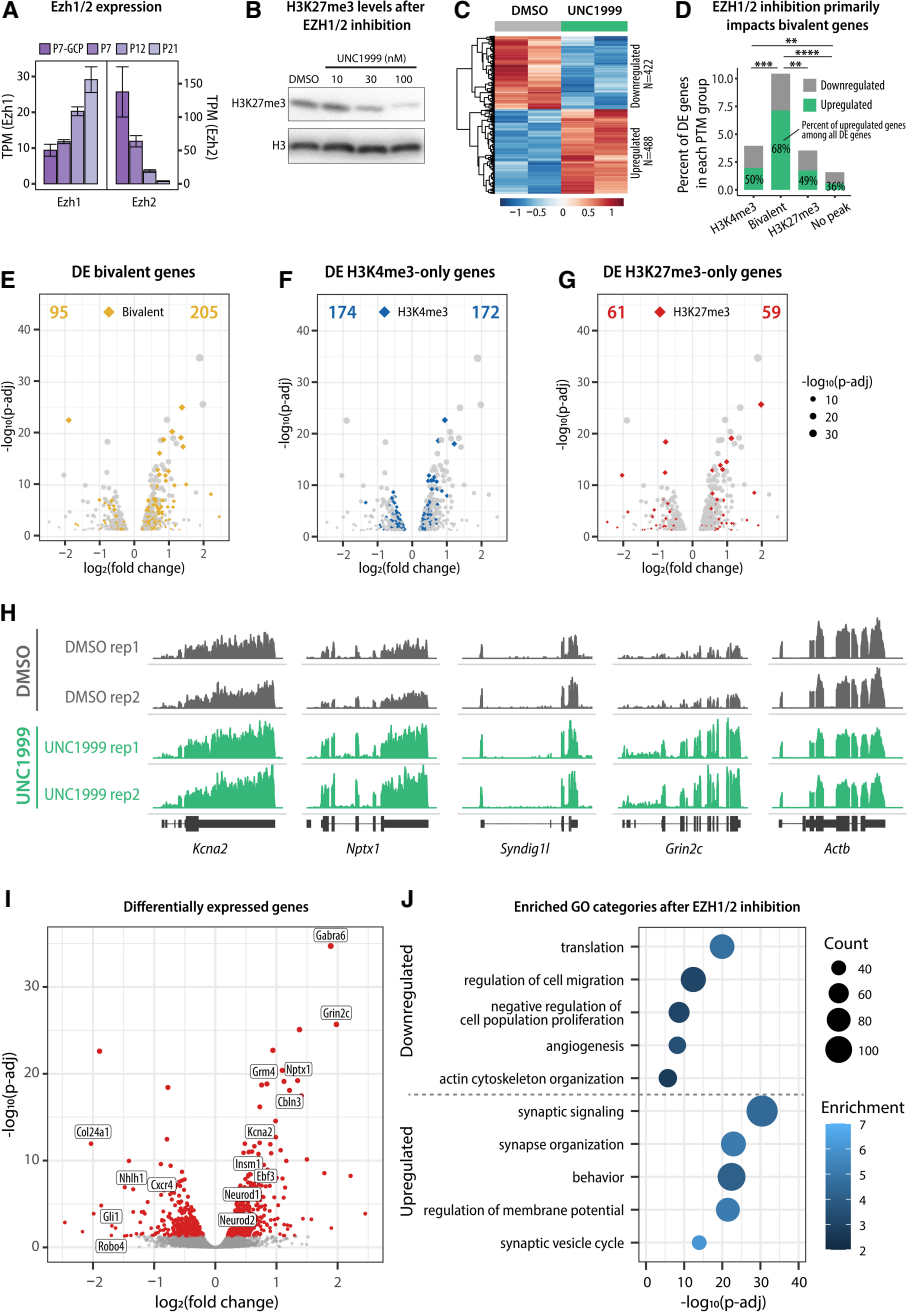
The effect of EZH1/2 inhibition on gene expression in cultured GCs. (*A*) Developmental expression of H3K27 methyltransferases *Ezh1* and *Ezh2*, based on TRAP RNA-seq (*n* = 4–5/group). (*B*) Immunoblot detection of H3K27me3 levels in GCs cultured for 5 d in vitro (DIV) in the presence of vehicle control (DMSO) or increasing concentrations of EZH1/2 inhibitor UNC1999 (in nanomolar). H3 was used as a loading control. (*C*) Heat map depicting differentially expressed genes in response to EZH1/2 inhibition after treatment with 100 nM UNC1999, compared with DMSO. (*D*) The percent of genes in each PTM category (based on P7 GCP ChIP-seq) that were up-regulated or down-regulated in cultured GCs in response to UNC1999 treatment compared with DMSO. More than 10% of all bivalent genes were differentially expressed, and the differentially expressed bivalent genes were significantly more likely to be up-regulated than down-regulated. Statistical analysis indicates the difference between the distribution of up-regulated and down-regulated genes between each PTM group. Pairwise Fisher test was performed using the rstatix package, and *P*-values were corrected for multiple comparisons using the FDR method. (**) *P* < 0.01, (***) *P* < 0.001, (****) *P* < 0.0001. (*E*–*G*) Volcano plots depicting differentially expressed bivalent (*E*), H3K4me3-only (*F*), or H3K27me3-only (*G*) genes in response to UNC1999 treatment, compared with DMSO. The number of up-regulated or down-regulated genes is indicated at the *top* of the chart. (*H*) Genome browser representation of RNA-seq tracks of selected bivalent genes at 5 DIV. *Actb* is shown as a control. (*I*) Volcano plot depicting differentially expressed genes (*P*-adj < 0.05, indicated with red) in response to UNC1999 treatment compared with DMSO. Up-regulated genes include several well-known marker genes of mature GCs, such as *Gabra6*, *Cbln3*, and *Grm4*. (*J*) Enriched GO categories of up-regulated and down-regulated genes after UNC1999 treatment compared with DMSO, identified using clusterProfiler package. The GO biological process categories were sorted by the adjusted *P*-value, and the top five enriched nonredundant categories are shown for each group.

We next asked whether perturbations in bivalency impact the developmental progression of immature GCs. To address this, we targeted H3K27me3 because our results suggested that H3K27me3 at bivalent loci is more dynamically regulated than H3K4me3 (Fig. 2D; Supplemental Fig. S2A). To reduce the levels of H3K27me3, we focused on the methyltransferases that generate this modification. H3K27 is methylated by the Polycomb repressive complex 2 (PRC2) that contains enhancer of Zeste 1 (EZH1) or EZH2 as the catalytic subunit. Both EZH1 and EZH2 are expressed throughout GC development, with EZH2 being highly expressed in proliferating GCPs and EZH1 being highly expressed in postmitotic cells (Fig. 6A). Importantly, even though EZH2 is the major H3K27 methyltransferase in proliferating cells, there is some evidence suggesting that EZH1 preferentially targets bivalent genes (Aoyama et al. 2018). Therefore, to ensure that our approach to reducing H3K27me3 impacts H3K27me3 at bivalent genes, we used a small molecule inhibitor, UNC1999, that inhibits both EZH1 and EZH2 (Konze et al. 2013).

To investigate how the reduction in H3K27me3 impacts gene expression in developing GCs, we used a well-established model of cultured GCs (Hatten 1985). Cultured GC progenitors isolated from P6–P7 cerebella exit the cell cycle within 24 h (Tomoda et al. 1999), after which they differentiate, migrate, and begin maturation over 4–5 d in vitro (DIV) (Edmondson and Hatten 1987; Solecki et al. 2004). To establish how gene expression in cultured GCs corresponds to in vivo GC development, we first determined the developmental gene expression pattern of cultured GCs at 0, 2, and 5 DIV using RNA-seq. We identified the highest expressed genes at each time point (Supplemental Fig. S4A,B) and performed GO analysis to identify enriched biological processes. We found that categories enriched in the 0 DIV transcriptome were associated with proliferation and DNA repair, categories enriched at 2 DIV were associated with synaptic signaling and neuron projection morphogenesis, and categories enriched at 5 DIV were associated with regulation of membrane potential (Supplemental Fig. S4C; Supplemental Table S2). These results were in line with the in vivo transcriptomics analysis (Fig. 1C) and suggested that the 0, 2, and 5 DIV time points correspond to GC proliferation, differentiation, and maturation, respectively.

Next, we treated cultured GCs with UNC1999, an inhibitor of EZH1 and EZH2, to evaluate the outcome of EZH1/2 inhibition on GC development. H3K27me3 levels were reduced after treatment with increasing concentrations of UNC1999 for 5 DIV compared with vehicle (DMSO) (Fig. 6B; Supplemental Fig. S5A), and treatment with 100 nM UNC1999 for 5 DIV was used in subsequent experiments. Compared with DMSO, UNC1999 did not induce DNA damage or cell death, as determined with γH2AX and active Casp3 staining, respectively (Supplemental Fig. S5A–D). In total, we identified 488 up-regulated genes and 422 down-regulated genes in response to UNC1999 treatment (Fig. 6C; Supplemental Table S3). While EZH1/2 inhibition was expected to reduce H3K27me3 levels at both bivalent and H3K27me3-only promoters, we found that the expression of bivalent genes was significantly more impacted by EZH1/2 inhibition than the expression of H3K4me3-only or H3K27me3-only genes (Fig. 6D–G). The majority of differentially expressed bivalent genes were up-regulated after EZH1/2 inhibition (Fig. 6D,H), consistent with the primary effect of removing a repressive modification from these genes. Remarkably, some of the most significantly up-regulated genes included classical markers of mature GCs, including *Gabra6*, *Neurod1*, *Cbln3*, *Grm4*, and *Grin2c*, as well as the bivalent TFs *Insm1*, *Neurod2*, and *Ebf3* identified above (Fig. 6I). Subsequent GO analysis of differentially expressed genes confirmed that down-regulated genes included categories involved in cell growth and migration, whereas genes up-regulated with UNC1999 treatment were involved in mature neuron function (Fig. 6J; Supplemental Table S2). The up-regulation of maturation-related genes was confirmed by RBFOX3/NeuN immunostaining (Supplemental Fig. S6A,B). Therefore, these results suggested that GCs cultured in the presence of an EZH1/2 inhibitor down-regulate migration-related genes and up-regulate genes involved in neuronal maturation.

**Figure 7.**
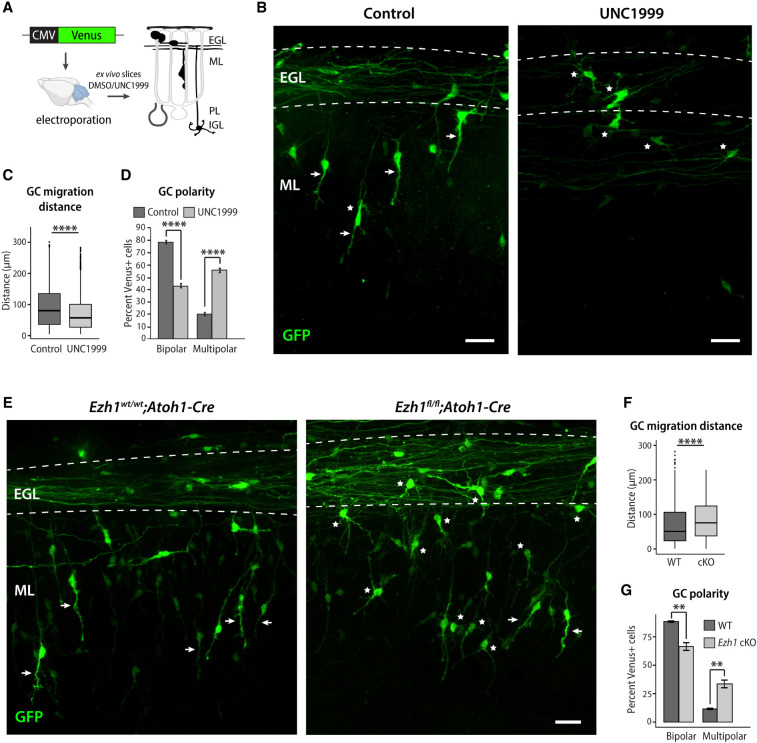
H3K4me3/H3K27me3 bivalency regulates the timing of GC neuronal maturation. (*A*) Schematic of the experimental design. A plasmid encoding the Venus fluorophore was electroporated into the cerebellum, and organotypic cerebellar slices were made and cultured in the presence of vehicle control (DMSO) or 200 nM UNC1999 for 60 h. The morphology and subcortical localization of GCs were evaluated to identify GCs at different stages of development. (*B*) Images of electroporated GCs in ex vivo cerebellar slices treated with vehicle control (DMSO) or UNC1999 for 60 h. Control cells exhibit a bipolar morphology typical of migrating GCs, with a leading process oriented in the direction of migration (white arrows) and few multipolar cells (white asterisks). In contrast, cells treated with UNC1999 displayed inhibited migration and multipolar morphology. Scale bar, 25 µm. (*C*) GC migration distance after vehicle control (DMSO) or UNC1999 treatment, measured as the distance of the cell soma to the outer edge of the parallel fiber axons. Each replicate included slices from two to three P8 mouse pups per condition. GC migration distance was quantified from four replicates for a total of 868 cells (DMSO) or 2334 cells (UNC1999). (*D*) Quantification of the percentage of bipolar and multipolar GCs out of all Venus-expressing cells in the molecular layer. Each replicate included slices from two to three postnatal P8 mouse pups per condition. For the vehicle control (DMSO) condition, regions of interest were imaged from five replicates for a total of 593 cells. For the UNC1999 condition, regions of interest were imaged from four replicates for a total of 880 cells. (*E*) Images of electroporated GCs in ex vivo cerebellar slices prepared from wild-type (*Ezh1*^*wt/wt*^;*Atoh1-Cre*) and *Ezh1* cKO (*Ezh1*^*fl/fl*^;*Atoh1-Cre*) mice. Similar to UNC1999-treated GCs, *Ezh1* cKO cells display an increased number of multipolar GCs (white asterisks) and fewer migrating GCs with a bipolar morphology (white arrows). Scale bar, 25 µm. (*F*) GC migration distance in wild-type and *Ezh1* cKO cells. Distances were measured from three P8 mouse pups per genotype. GC migration distance was quantified from a total of 981 cells (wild type) or 1066 cells (*Ezh1* cKO). (*G*) Quantification of the percentage of bipolar and multipolar GCs out of all Venus-expressing cells in the molecular layer. Cells were counted from three P8 mouse pups per genotype. Regions of interest were imaged from slices prepared from each mouse pup for each condition for a total of 378 cells (wild type) or 512 cells (*Ezh1* cKO). Student's *t*-test. (**) *P* < 0.01, (****) *P* < 0.0001.

To directly observe the effect of EZH1/2 inhibition on GC development, we treated ex vivo slices of P8 cerebellum with UNC1999 or vehicle control (DMSO) (Fig. 7A). Prior to UNC1999 or vehicle treatment, we electroporated P8 cerebella with a plasmid expressing a Venus fluorophore to allow visualization of the progression of labeled GCs through axon outgrowth, glial-guided migration and dendrite extension in specific layers of the cerebellum (Govek et al. 2018). In this system, after 60 h ex vivo, GCs treated with the vehicle were located in the lower EGL, where they extended bipolar parallel processes, and in the molecular layer, where they exhibited a bipolar morphology with a single leading process typical of migrating cells and a trailing T-shaped axon (Fig. 7B, white arrows). In contrast, GCs treated with UNC1999 failed to enter the deeper aspects of the molecular layer (Fig. 7B). On average, the distance of migration, calculated by measuring the distance of the cell soma to the outer edge of the parallel fiber axons, was reduced by 25% after UNC1999 treatment compared with the vehicle control (Fig. 7C). Instead, a significant number of UNC1999-treated cells displayed a multipolar morphology typical of postmigratory, terminally differentiated GCs (Fig. 7B, white asterisks; Zhu et al. 2016), suggesting that EZH1/2 inhibition induced premature maturation. Quantification of the number of bipolar and multipolar GCs in the molecular layer revealed that UNC1999 treatment significantly decreased the percentage of bipolar GCs and increased the percentage of multipolar GCs (Fig. 7D). Therefore, by both morphological criteria and laminar position, UNC1999 treatment inhibited glial-guided migration of GCs and induced premature terminal differentiation. Together, these results suggest that disrupting bivalency in GCPs perturbs glial-guided neuronal migration, a key step in CNS development, and accelerates terminal differentiation.

*Ezh1* expression is up-regulated in postmitotic neurons (Fig. 6A), and EZH1 has been suggested to target bivalent genes (Aoyama et al. 2018). Therefore, we next studied whether EZH1 loss of function would recapitulate some or most gene expression and morphological changes observed with EZH1/2 inhibition. To that end, we crossed *Ezh1*^*fl/fl*^ mice (Hidalgo et al. 2012) with *Atoh1-Cre* mice to obtain *Ezh1*^*fl/fl*^;*Atoh1-Cre* mice (referred to as the *Ezh1* cKO mice) to delete the catalytic domain of EZH1 in GC progenitors from E9.5 onward. We cultured GCPs isolated from P7 *Ezh1* cKO mice and wild-type littermates and performed RNA-seq on DIV5. We then analyzed how the expression of genes differentially expressed after UNC1999 treatment changes with *Ezh1* deletion. We found that the expression of 71% of UNC1999 DE genes changed in the same direction (also up-regulated or down-regulated) in *Ezh1* cKO (Supplemental Fig. S7A), though the degree of gene expression changes was generally smaller in *Ezh1* cKO versus wild-type GCs compared with UNC1999 versus DMSO GCs (Supplemental Fig. S7A; Supplemental Table S4). To compare the pathways altered by UNC1999 and *Ezh1* cKO, we performed gene set enrichment analysis (GSEA) using the statistical value from DESeq2 analysis (Supplemental Table S2). Comparison of all significantly enriched gene sets showed that UNC1999 treatment and *Ezh1* deletion resulted in 97 shared pathways with a positive normalized enrichment score (NES) and 91 shared pathways with a negative NES in both (Supplemental Fig. S7B; Supplemental Table S2). Notably, the most significant pathways with positive NES with both UNC1999 treatment and *Ezh1* cKO were related to synaptic signaling and dendrite development (Supplemental Fig. S7C,D; Supplemental Table S2), which are markers of terminal differentiation.

We then evaluated GC glial-guided migration and polarity in ex vivo cerebellar slices prepared from *Ezh1* cKO mice and wild-type littermates (Fig. 7E). Similar to UNC1999-treated GCs, we observed a significant increase in the number of fine dendrites in *Ezh1* cKO GCs located in the molecular layer, which would normally only include cells with a single, leading process (Fig. 7E,G). The *Ezh1* cKO GCs also extended leading processes of variable length and morphology compared with control cells yet migrated into the molecular layer (Fig. 7E,F). Thus, the *Ezh1* cKO GCs shared morphological features with UNC1999-treated GCs, consistent with the enrichment of gene ontology pathways related to dendrite development and synaptic signaling (Supplemental Fig. S7C). Together, these results suggest that *Ezh1* deletion recapitulates key aspects of the morphological and gene expression changes observed with EZH1/2 inhibition.

### GCP bivalent domains are enriched at marker genes of other cerebellar neurons

Given the proposed role of bivalency in lineage specification, we also analyzed whether bivalency is required to maintain cell type-specific gene expression programs in proliferating GCPs and postmitotic neurons. To address this, we took advantage of published scRNA-seq data from adult mouse brains (Saunders et al. 2018), as well as the developmental lineage of cerebellar cells (Supplemental Fig. S8A; Vladoiu et al. 2019). First, we reasoned that if bivalency is required to control lineage-appropriate gene expression in proliferating GCPs, we would observe a higher proportion of bivalent genes among genes specific to closely related cell types (e.g., unipolar brush cells) compared with distant cell types (e.g., microglia) (Supplemental Fig. S8A). To test this possibility, we evaluated the proportion of bivalent promoters identified in P7 GCPs among marker genes of different types of cerebellar cells, identified based on scRNA-seq (Saunders et al. 2018). Consistent with our hypothesis, a high proportion (35%–50%) of marker genes of other cerebellar neurons were bivalent in proliferating GCPs, at a level that was significantly higher than expected genome-wide (Supplemental Fig. S8A). These results are consistent with the interpretation that bivalency maintains lineage-specific gene expression in committed neuronal progenitors.

To address the question of whether H3K27me3 and bivalency could repress the expression of other cell type marker genes in differentiated GCs, we analyzed how UNC1999 treatment impacts the expression of the identified marker genes in cultured GCs. We found that while GC-specific genes were significantly up-regulated by UNC1999 treatment, the expression of other cerebellar cell type marker genes was essentially unchanged (Supplemental Fig. S8B–D). These results demonstrate that in postmitotic GCs, the loss of H3K27me3 does not lead to an overexpression of genes specific to other, even closely related, cell types. In other words, by the time immature neuronal progenitors become postmitotic, the loss of bivalency and H3K27me3 has minimal impact on the expression of genes specific to other cell lineages.

## Discussion

Here we report that bivalency controls the gene expression programs required for glial-guided migration and maturation of an identified, well-studied CNS neuron: the cerebellar granule cell. Our studies using a genetically encoded probe for identifying bivalent domains demonstrate that bivalency exists in individual GCs. Our experiments using histone PTM and gene expression profiling demonstrate that bivalency regulates the expression of neuronal genes during GCP differentiation. In addition, inhibition of EZH1/2 inhibits glial-guided migration and accelerates dendrite formation, suggesting that bivalency regulates the timing of key steps in cerebellar GC differentiation. Therefore, these results demonstrate that the role of bivalency extends beyond the control of lineage development and pertains to the regulation of developmental progression of committed CNS progenitor cells.

Our study illustrates the importance of functional assays combined with molecular studies to define the relationship between gene expression and key biological processes. The use of a genetically encoded sensor with histone methylation reader domains for detecting bivalent nucleosomes is a powerful method for testing whether individual GCs have bivalent domains as opposed to bivalency being an averaged feature of GCs. Our data revealed discrete subnuclear focal areas of fluorescent labeling, consistent with the conclusion that individual GCs contain bivalent domains. These results were in line with a prior study using the cMAP3 bivalency probe, which demonstrated that GCPs organize their poised chromatin in defined locations of H3.3-free euchromatin (Hoffman et al. 2020). The methodology does not provide quantitative details on the extent of bivalency in the positive domains, as discussed in Delachat et al. (2018). However, a key finding from our experiments was that the fluorescent subnuclear foci were evident in proliferating GCPs and not in postmitotic GCs. This finding was consistent with the molecular data showing that bivalency peaked at P7, the time of maximal GCP proliferation.

Bivalent genes were primarily neuronal genes that were expressed at low to intermediate levels and were differentially expressed during GC differentiation and maturation. Of the genes identified as bivalent in the current study, of particular interest were several TFs known to be essential for specifying GC identity and differentiation. The finding that these genes also had the widest H3K4me3 peaks is consistent with prior studies showing that broad H3K4me3 domains mark genes that determine cell identity (Benayoun et al. 2014). Taken together, these findings confirmed the presence of bivalency in individual GCPs and implicated bivalency in the establishment of GC identity, the fine-tuning of gene expression levels, and the control of gene expression dynamics during neuronal development.

The role of chromatin dynamics in regulating gene expression demonstrated in the current study is consistent with our prior finding that the expression of *Tet* genes controls the expression of axon guidance and ion channel genes, underlying the formation of dendrites and synaptic connections in GCs (Zhu et al. 2016). TET enzymes catalyze the conversion of DNA 5-methylcytosine to 5-hydroxymethylcytosine, a modification that during terminal differentiation accumulates in neuronal chromatin in a cell type-specific manner and correlates with active transcription (Kriaucionis and Heintz 2009; Gabel et al. 2015; Mellén et al. 2017; Stoyanova et al. 2021). In addition to regulating gene expression during GC maturation, TET activity is also required for the terminal differentiation of Purkinje cells (Stoyanova et al. 2021) and activity-dependent gene expression in hippocampal and cortical neurons (Rudenko et al. 2013). It is noteworthy that in mouse ESCs, TET1 directly interacts with PRC2 (Neri et al. 2013) and binds bivalent promoters (Villaseñor et al. 2020), where it regulates the levels of DNA methylation (Gu et al. 2018; Verma et al. 2018; Xiang et al. 2020). Thus, a possible model emerges that in immature neurons, bivalency could mark the promoters that become targets of TET enzymes during neuronal differentiation, thereby orchestrating the formation of the regulatory chromatin landscape in mature neurons. The detailed characterization of the interactions between bivalency and DNA hydroxymethylation during neuronal development and circuitry formation will be important to resolve this question.

The importance of bivalency on GCP gene expression and development was revealed by treating ex vivo slices of cerebellar cortex and cultured GCs with UNC1999, a selective inhibitor for the H3K27 methyltransferases EZH1 and EZH2. Strikingly, UNC1999 inhibited migration and promoted dendrite extension of GCs in the organotypic slice assay. This result was supported by molecular studies in cultured GCs showing that after treatment with UNC1999, GO pathways for migration were down-regulated, whereas those for synapse formation were up-regulated. Although inhibition of EZH1 and EZH2 was expected to alter both bivalent and H3K27me3 monovalent genes, we found that UNC1999 predominantly affected the expression of bivalent genes. These results were consistent with our observations that the levels of H3K27me3 are lower at bivalent genes than at H3K27me3-only genes and that the ratio of H3K4me3 to H3K27me3 at bivalent domains correlates with gene expression levels. Our findings further show that inhibiting both EZH1 and EZH2 using UNC1999 induced gene expression and polarity changes that were recapitulated by a loss of EZH1. These results are consistent with a previous study suggesting that bivalent genes are core targets of EZH1 (Aoyama et al. 2018). Further studies will be required to fully analyze the targets and function of EZH1 and EZH2 in the regulation of H3K27me3 and bivalency in developing neurons. Moreover, it remains to be determined how H3K4 methyltransferases (in particular MLL2/KMT2B) (Hu et al. 2013) and histone demethylases regulate bivalency and the timing of GC developmental progression.

The finding that EZH1/2 inhibition accelerates terminal maturation but inhibits glial-guided migration was unexpected given that prior reports have shown that stimulating early steps in GCP differentiation, such as by inhibiting the transcription factor ZEB1 (Singh et al. 2016) or overexpressing NEUROD1 (Hanzel et al. 2019), induces polarization required for the onset of migration and promotes glial-guided migration. In contrast, inhibiting bivalency prematurely induces the gene expression program associated with mature neurons, as evident from the up-regulation of synaptic genes and increased percentage of multipolar GCs after treatment with UNC1999. The accelerated maturation was accompanied by inhibited migration, as GCs treated with UNC1999 down-regulated genes involved with migration and failed to migrate into the IGL. Our results are therefore consistent with the model that bivalency is required to poise the expression of genes involved in terminal differentiation until migration is completed, and premature induction of neuronal maturation blocks glial-guided migration.

While bivalency peaked at P7 during GCP proliferation, we also observed bivalent domains in mature GCs, leaving open the question of the role of bivalency in mature neurons. Although most bivalent genes resolved to monovalent during GC maturation, we identified >1000 bivalent genes that either remained bivalent or became de novo bivalent in mature GCs. Our experiments using the EZH1/2 inhibitor do not support the hypothesis that bivalency is required to prevent transdifferentiation of postmitotic neurons into other types of cells. However, we found that genes that remained bivalent in mature GCs were involved in developmental processes and included growth factors, axon guidance genes, and transcription factors important for GCP specification in development (*Atoh1*, *Gli3*, and *Mycn*). While the present study did not examine the function of bivalency in mature neurons that are integrated into the cerebellar circuitry, it will be of interest to determine whether bivalency at these genes allows their reactivation; for example, as a response to neuronal damage. Another point to raise in this context is that *Atoh1*, *Gli3*, and *Mycn* are all up-regulated in a subtype of medulloblastoma that originates from GCs. Therefore, dysregulation of bivalent genes in postmitotic neurons could potentially have relevance in cancer pathogenesis.

The model that emerges from this study is that bivalency controls the timing of the progression of steps that define the characteristics of specific CNS neurons. In the future, it will be important to examine the role of other histone PTMs in regulating the developmental progression of neuronal cell types of the CNS. Understanding how epigenetic mechanisms control the functional maturation of neurons will inform future studies on the formation of CNS circuitries and the emergence of neurodevelopmental disorders.

## Materials and methods

### Animals

All animal experiments were conducted in accordance with the United States Animal Welfare Act and the National Institutes of Health's policy to ensure proper care and use of laboratory animals for research and under established guidelines and supervision by the Institutional Animal Care and Use Committee (IACUC) of the Rockefeller University. Mice were housed in accredited facilities of the Association for Assessment of Laboratory Animal Care (AALAC) in accordance with the National Institutes of Health guidelines. All efforts were made to minimize the suffering of animals. Both male and female animals were included in the study. C57Bl/6J mice were purchased from the Jackson Laboratories. *Tg(NeuroD1-Egfp-L10a)* and *Tg(Atoh1-Egfp-L10a)* TRAP mice were a gift from Dr. Nathaniel Heintz. *Ezh1*^*fl/fl*^ mice were a gift from Dr. Anne Schaefer. *Atoh1-Cre* mice were a gift from Dr. David Rowitch and Dr. Alex Joyner. The mice were group-housed in a specific pathogen-free stage with ad libitum access to food and water under a 12-h light/dark cycle (on 7:00 a.m./off 7:00 p.m.). Bedding and nest material were changed weekly. Where appropriate, ARRIVE (animal research: reporting of in vivo experiments) guidelines were followed for reporting animal research.

### Plasmids

The pCIG2-Venus plasmid was described in Govek et al. (2018). A plasmid encoding the cMAP3 bivalency probe was a gift from David Solecki (Delachat et al. 2018; Hoffman et al. 2020). The cMAP3 sequence, consisting of Polycomb chromodomain (PCD), Emerald fluorophore, and transcription factor TFIID subunit 3 plant homeodomain (TAF3), was subcloned into the pCIG2 vector. The pCIG2-cMAP3 plasmid was further modified to coexpress the tdTomato fluorophore under an IRES.

### Isolation and labeling of nuclei

Nucleus isolation was adapted from Xu et al. (2018) with modifications. P12 and P21 cerebella were dissected from C57Bl/6J mice and either snap-frozen or processed fresh. For isolation of nuclei, four to six cerebella were pooled. The tissue was homogenized in homogenization buffer (0.25 M sucrose, 150 mM KCl, 5 mM MgCl_2_, 20 mM Tricine-KOH at pH 7.8, 0.15 mM spermine, 0.5 mM spermidine, EDTA-free protease inhibitor cocktail, PhosSTOP, 1 mM DTT) by 30 strokes with loose (A) pestle followed by 30 strokes with tight (B) pestle in a glass Dounce homogenizer on ice. The homogenate was supplemented with 0.92 vol of iodixanol solution (50% iodixanol/optiprep, 150 mM KCl, 5 mM MgCl_2_, 20 mM Tricine at pH 7.8, 0.15 mM spermine, 0.5 mM spermidine, EDTA-free protease inhibitor cocktail, PhosSTOP, 1 mM DTT) and laid on a 27% iodixanol cushion. Nuclei were pelleted by centrifugation at 17,200*g* for 30 min at 4°C in a standard tabletop centrifuge. The pellet was resuspended in the homogenization buffer and passed through a cell strainer. Resuspended nuclei were fixed with 1% formaldehyde for 7 min at room temperature and quenched with 0.125 M glycine for 5 min at room temperature. Nuclei were pelleted by centrifugation at 1000*g* for 5 min at 4°C and washed once with the homogenization buffer and once with wash buffer (3% BSA, 0.05% Triton X-100 in PBS). Nuclei were blocked with block buffer (6% BSA, 0.05% Triton X-100 in PBS) for 1 h at room temperature and incubated with anti-NeuN (1:500–1:1000; Millipore MAB377, RRID:AB_2298772) and anti-NeuroD1 (1:100; Santa Cruz Biotechnology sc-1084, RRID:AB_630922) antibodies overnight at 4°C. The nuclei were washed three times with wash buffer at 1000*g* centrifugation for 5 min at 4°C in between, incubated with secondary antibodies (1:500; Alexa fluor 488 donkey antimouse IgG [Invitrogen A21202, RRID AB_141607] and Alexa fluor 555 donkey antirabbit IgG [Invitrogen A31572, RRID AB_162543]) for 30 min at room temperature, and washed three times with wash buffer. Primary and secondary antibodies were diluted in the block buffer.

### Flow cytometry

Labeled nuclei were resuspended in 1% BSA and 10 mM HEPES in PBS and incubated with DyeCycle Ruby (Thermo Fisher Scientific V10309) for 30 min at room temperature protected from light. Sorting was carried out at the Flow Cytometry Resource Center at Rockefeller University using a BD FACSAria cell sorter using the 70-µm nozzle and 488-, 561-, and 635-/640-nm lasers. Samples were gated using DyeCycle Ruby to identify singlets, followed by the sorting of the NeuN^+^/NeuroD1^+^ population. Sorted nuclei were pelleted by centrifugation, resuspended in DPBS to divide into aliquots of 10 × 10^6^ nuclei/tube, centrifuged again, and stored at −80°C. In these experiments, 95%–98% of singlets were NeuN^+^/NeuroD1^+^, and the average yield was 6 × 10^6^ to 10 × 10^6^ nuclei per one P12 or P21 cerebellum. The specificity of the primary antibodies and the gating strategy was confirmed using *Tg(NeuroD1-Egfp-L10a)* mice that express EGFP specifically in postmitotic GCs of the cerebellum.

### GCP purification

P7 GCPs were isolated from C57Bl/6J mice as previously described (Hatten 1985). Briefly, isolated cerebella were dissected in ice-cold CMF-PBS and dissociated with trypsin-DNase I for 5 min at 37°C, followed by centrifugation at 700*g* for 5 min at 4°C. Trypsin–DNase I was removed, and the cells were triturated in DNase-CMF-PBS 10 times with a transfer pipette, 10 times with a fine fire-polished Pasteur pipette, and 10 times with an extrafine Pasteur pipette. The cell homogenate was then centrifuged at 700*g* for 5 min at 4°C, and the cell pellet was resuspended in DNase–CMF–PBS. The cells were subjected to Percoll gradient sedimentation to enrich for proliferating GCPs and subsequently preplated for 15–30 min on a Petri dish and 1–2 h on a tissue culture dish to remove glia and fibroblasts. The medium was collected and centrifuged at 700*g* for 5 min at 4°C to pellet the GCPs. Cells dedicated for ChIP were subsequently resuspended in DPBS, fixed with 1% formaldehyde for 7 min at room temperature, quenched with 0.125 M glycine for 5 min, washed once with DPBS, and frozen in aliquots of 5 × 10^6^ to 10 × 10^6^ cells.

#### GCP cultures and UNC1999 treatment

GCPs isolated after preplating were subsequently plated on a 24-well plate or 16-well chamber slides (Thermo Scientific Nunc Labtek 12-565-110N) precoated with 0.1 mg/mL poly-D-lysine (Sigma P1024) and Matrigel in granule cell medium (BME [Life Technologies 21010-046]; 2 mM L-glutamine [Gibco 25030-016], 1× Pen-Strep [Gibco 15140-015], 0.9% glucose [Sigma G8769], 10% horse serum [heat-inactivated; Gibco 16050-122], 5% fetal bovine serum [heat-inactivated; Gibco 26140-079]) at a final concentration of 2.5 × 10^6^ cells in 600 µL. A final concentration of 10–100 nM UNC1999 (Tocris Biosciences) or an equivalent volume of DMSO was added to the culture medium starting at 0 DIV. One-half of culture medium was replaced with fresh medium plus DMSO or UNC1999 daily until 5 DIV. In experiments using *Ezh1*^*fl/fl*^ × *Atoh1-Cre* mice, GCPs were isolated from individual *Ezh1*^*wt/wt*^;*Atoh1-Cre* and *Ezh1*^*fl/fl*^;*Atoh1-Cre* mice from two independent litters of *Ezh1*^*wt/fl*^;*Atoh1-Cre* × *Ezh1*^*wt/fl*^;*Atoh1-Cre* breedings (*n* = 2 wild-type and *n* = 2 *Ezh1* cKO mice in the first litter, and *n* = 3 wild-type and *n* = 2 *Ezh1* cKO mice in the second litter), plated, and cultured until 5 DIV. Cells were collected by scraping, pelleted by centrifugation, and stored at −80°C until processing for RNA and protein isolation.

### RNA isolation

RNA from cultured GCs was isolated using RNeasy micro kit (Qiagen 74004) with on-column DNA digestion. RNA quality was analyzed with the Bioanalyzer RNA 6000 Pico kit (Agilent) prior to library preparation.

### Protein isolation

Nuclear and cytoplasmic protein fractions were isolated using subcellular fractionation protocol. Briefly, nuclei were isolated in subcellular fractionation buffer (20 mM HEPES, 10 mM KCl, 2 mM MgCl_2_, 1 mM EDTA, 1 mM EGTA, supplemented with 0.5 mM DTT, 0.2 mM PMSF, protease and phosphatase inhibitors), incubated for 15 min on ice, passed through a 27-gauge needle three to five times, incubated for another 20 min on ice, and pelleted at 3000 rpm for 5 min at 4°C. Supernatant containing the cytoplasmic fraction was stored at −80°C. The pellet containing the nuclei was then resuspended in TBS containing 0.1% SDS and sonicated using Bioruptor Pico (Diagenode) at 30 sec on/30 sec off for 10 cycles. Insoluble fraction was pelleted by centrifugation at 13,000 rpm for 10 min at 4°C, and supernatant containing nuclear proteins was stored at −80°C.

### Immunoblotting

Samples were run on 16% Tris-glycine SDS-PAGE gels (Invitrogen) and transferred to 0.2-µm nitrocellulose membranes. The membranes were blocked in 5% milk in TBST and incubated with antibody solutions in 2.5% milk, with TBST washes in between. Proteins were visualized using Immobilon ECL (Invitrogen) and D1001 KwikQuant imager (Kindle Biosciences). The following antibodies were used: rabbit anti-H3K27me3 (1:300; Cell Signaling Technology 9733, RRID:AB_2616029), rabbit anti-H3 (1:5000; Abcam ab1791, RRID:AB_302613), and goat antirabbit IgG (H + L)-HRP conjugate (Bio-Rad 170-6515, RRID:AB_11125142).

### Chromatin immunoprecipitation (ChIP)

#### Lysis and sonication

Fixed GCPs were thawed on ice, resuspended in cell lysis buffer (50 mM HEPES-KOH at pH 7.5, 140 mM NaCl, 1 mM EDTA, 10% glycerol, 0.5% NP-40, 0.25% Triton X-100, supplemented with 0.5 mM DTT, 0.2 mM PMSF, protease and phosphatase inhibitors) at 1 mL per 10 × 10^6^ cells, and incubated for 10 min at 4°C using end-over-end rotation. Nuclei were isolated by centrifugation at 1350*g* for 5 min at 4°C. Fixed and sorted nuclei from P12 and P21 mice were thawed on ice. GCP nuclei and nuclei isolated with FACS were subsequently resuspended in nucleus lysis buffer (50 mM Tris-HCl at pH 8, 10 mM EDTA, 1% SDS, supplemented with 0.5 mM DTT, 0.2 mM PMSF, protease and phosphatase inhibitors) at 140 µL per 10 × 10^6^ nuclei and incubated for 10 min at room temperature using end-over-end rotation. Lysates were passed through a 27-gauge needle two to three times and sonicated using a Covaris E220 focused ultrasonicator in an AFA fiber preslit Snap-Cap microTUBE at 140 W peak power, 10% duty factor, and 200 cycles/burst for 1 min 50 sec for P7 GCP nuclei or 2 min 10 sec for P12 and P21 GC nuclei. Triton X-100 was added at a final concentration of 1%, and insoluble chromatin was pelleted by centrifugation at 13,000 rpm for 10 min at 4°C. Five percent of chromatin was used for determining sonication efficiency. Samples were stored at −80°C until processing.

#### ChIP

Sonicated chromatin was diluted 10× with dilution buffer (1 mM Tris-HCl at pH 8.0, 167 mM NaCl, 0.1 mM EDTA, 0.5% sodium deoxycholate, 1% NP-40, supplemented with 0.5 mM DTT, 0.2 mM PMSF, protease and phosphatase inhibitors). In a subset of experiments, *Drosophila* chromatin (1 ng per 1 µg of chromatin; Active Motif 53083) was added to the samples. Five percent of chromatin was used for input control. Protein A Dynabeads (Invitrogen 10002D) were coated with antibodies (rabbit anti-H3K27me3 [Cell Signaling Technology 9733, RRID:AB_2616029] and rabbit anti-H3K4me3 [Active Motif 39159, RRID:AB_2615077]) in 0.5% BSA in PBS for 2 h at 4°C using end-over-end rotation and washed three times with 0.5% BSA in PBS. Antibody-coated beads were added to the samples and incubated rotating overnight at 4°C. The beads were then washed eight times with wash buffer (50 mM HEPES-KOH at pH 7.6, 500 mM LiCl, 1 mM EDTA, 1% NP-40, 0.7% sodium deoxycholate) and once with TE containing 50 mM NaCl. The chromatin was eluted from the beads with elution buffer (50 mM Tris-HCl at pH 8.0, 10 mM EDTA, 1% SDS) for 30 min with shaking at 65°C, and samples were incubated overnight at 65°C to reverse cross-links. RNA was digested with 5 µg/mL DNase-free RNase (Roche 11119915001) for 1 h at 37°C, and proteins were digested with 0.2 mg/mL proteinase K (Thermo Scientific EO0491) for 1 h at 55°C. DNA was purified using PCR purification kit (Qiagen 28104). ChIP efficiency prior to sequencing was evaluated using ChIP qPCR (data not shown).

### ChIP sequencing

ChIP-seq libraries were prepared using the TruSeq ChIP library preparation kit (Illumina IP-202-1012 or IP-202-1024) or the NEBNext Ultra II DNA library preparation kit for Illumina (E7645S). The quality of the sequencing libraries was evaluated using the Agilent 2200 TapeStation with D1000 High-Sensitivity ScreenTape. The samples were sequenced at the Rockefeller University Genomics Resource Center using a NextSeq 500 sequencer (Illumina) to obtain 75-bp single-end reads.

### Translating ribosome affinity purification (TRAP)

#### Antibody binding

TRAP was performed as previously described (Doyle et al. 2008; Heiman et al. 2008). For each reaction, 300 µL of Streptavidin MyOne T1 Dynabeads (Invitrogen 65601) was washed five times with 1× PBS and incubated with 120 µg of biotinylated protein L (reconstituted at 1 µg/µL in PBS; Pierce 29997) in 1× PBS in a total volume of 1 mL for 35 min at room temperature using end-over-end rotation. The beads were then washed five times with 3% IgG and Protease-free BSA (JacksonImmuno 001-000-162) in 1× PBS and subsequently incubated with 50 µg each of 19C8 and 19F7 anti-GFP monoclonal antibodies (Memorial Sloan-Kettering Monoclonal Antibody Facility) in 500 µL of 0.15 M KCl TRAP wash buffer (10 mM HEPES-KOH at pH 7.4, 5 mM MgCl_2_, 150 mM KCl, 1% NP-40, supplemented with 100 µg/mL cycloheximide [Millipore Sigma C7698-1g] in methanol, 0.5 mM DTT [Thermo Fisher Scientific R0861], 20 U/mL RNasin [Fisher Scientific PR-N2515]) just before use for 30 min using end-over-end rotation. After antibody binding, the beads were washed three times with 0.15 M KCl TRAP wash buffer and resuspended in 0.15 M KCl TRAP wash buffer, and each reaction was supplemented with 30 mM DHPC (Avanti 850306P).

#### Brain lysate preparation

Cerebella from P7, P12, or P21 *Tg(NeuroD1-Egfp-L10a)* mice were dissected and placed in TRAP dissection buffer (2.5 mM HEPES-KOH at pH 7.4, 35 mM glucose, 4 mM NaHCO_3_ in 1× HBSS, supplemented with 100 µg/mL cycloheximide) until ready for homogenization. The tissue was homogenized in chilled TRAP lysis buffer (10 mM HEPES-KOH at pH 7.4, 5 mM MgCl_2_, 150 mM KCl, supplemented with 0.5 mM DTT, 100 µg/mL cycloheximide, protease inhibitor cocktail [Sigma 11836170001], 40 U/mL RNasin, 20 U/mL Superasin [Thermo Fisher Scientific AM2694] just before use) using a motor-driven Teflon-Glass homogenizer at 900 rpm and 12 strokes. The homogenate was centrifuged at 2000*g* for 10 min at 4°C, and 3% of the supernatant was set aside as an input. The rest of the supernatant was mixed with NP-40 to a final concentration of 1% and with DHPC to a final concentration of 30 mM and incubated for 5 min on ice. Subsequently, the samples were centrifuged at 20,000*g* for 10 min at 4°C, and the supernatant was used for immunoprecipitation.

#### GCP preparation for TRAP

GCPs from P7 *Tg(Atoh1-Egfp-L10a)* mice were isolated using Percoll gradient centrifugation as described above. Pooled GCPs from two to three mice were homogenized in TRAP lysis buffer and processed in parallel with brain lysates described above. Since the isolated cells were enriched for GCPs, input was not collected.

#### Immunoprecipitation

The samples were incubated with GFP-conjugated beads overnight at 4°C with end-over-end rotation. Subsequently, the beads were washed four times in 0.35 mM KCl TRAP wash buffer (10 mM HEPES-KOH at pH 7.4, 5 mM MgCl_2_, 350 mM KCl, 1% NP-40, supplemented with 100 µg/mL cycloheximide, 0.5 mM DTT, 20 U/mL RNasin) just before use and resuspended in 100 µL of RLT buffer from the RNeasy micro kit (Qiagen 74004) supplemented with 1% β-mercaptoethanol. The resuspended beads were incubated for 10 min at room tempterature and placed on a magnet, and the supernatant containing the RNA was collected and purified using the RNeasy micro kit. RNA integrity was determined using Bioanalyzer RNA 6000 Pico kit (Agilent) prior to library preparation.

### RNA sequencing

RNA-seq libraries were prepared using the NEBNext Ultra II RNA library preparation kit for Illumina (NEB E7770S) in conjunction with the NEBNext poly(A) mRNA magnetic isolation module (NEB E7490) and NEBNext multiplex oligos for Illumina (NEB E7335 and E7500). For library preparation, we used 10 ng of TRAP RNA from *Tg(Atoh1-Egfp-L10a)* mice, 800 ng of TRAP RNA from *Tg(NeuroD1-Egfp-L10a)* mice, or 50–500 ng of total RNA from cultured GCs. Input samples for each developmental age in the TRAP experiment were pooled in equal amounts to yield one input per time point. The quality of the sequencing libraries was evaluated using the Agilent 2200 TapeStation with D1000 High-Sensitivity ScreenTape. The samples were sequenced at the Rockefeller University Genomics Resource Center using a NextSeq 500 sequencer (Illumina) to obtain 75-bp single-end reads.

### Bioinformatics analysis

Sequence and transcript coordinates for mouse mm10 UCSC genome and gene models were retrieved from the Bioconductor Bsgenome.Mmusculus.UCSC.mm10 (version 1.4.0) and TxDb.Mmusculus.UCSC.mm10.knownGene (version 3.4.0) libraries, respectively.

#### ChIP-seq

ChIP-seq reads were mapped using the Rsubread package's align function (version 1.30.6) (Liao et al. 2019). Peak calls were made with HOMER (version 4.11; style histone, size = 1000, minDist = 2500) (Heinz et al. 2010). Consensus peaks were determined to be peaks that were found in the majority of replicates. Peaks were annotated and genome distribution was determined using the ChIPseeker package (version 1.30.3) (Yu et al. 2015). Bivalent genes were those assessed to contain overlapping H3K27me3 and H3K4me3 peaks that overlap with the TSS (±500 bp). We note that the peaks of H3K27me3 and H3K4me3 signals at identified bivalent regions did not always coincide. Pairwise comparisons between developmental time points were made using DEseq2 (version 1.34.0) (Love et al. 2016) using counts from TSSs, with significant genes considered as *P*-adj < 0.05. DESeq2 was also used to derive the bivalency ratio through a pairwise comparison between H3K27me3 and H3K4me3 at TSSs. For the analysis of broad H3K4me3 domains, peaks with a distance of <1 kb were merged, ranked by width, and divided into broad and typical peaks using the elbow point method as in Whyte et al. (2013). Normalized, fragment-extended signal bigWigs were created using the rtracklayer package (version 1.40.6) (Lawrence et al. 2009) and then visualized and exported using Gviz (version 1.38.4). Heat maps and metaplots were generated with profileplyr (version 1.10.2; https://www.bioconductor.org/packages/release/bioc/html/profileplyr.html).

#### RNA*-*seq

RNA-seq reads were mapped using Rsubread. Transcript expressions were calculated using the Salmon quantification software (version 0.8.2) (Patro et al. 2017), and gene expression levels as TPMs and counts were retrieved using Tximport (version 1.8.0) (Love et al. 2016). Normalization and rlog transformation of raw read counts in genes, PCA, and differential gene expression analysis were performed using DESeq2. Additional sample-to-sample variability assessment was made with heat maps of between-sample distances using pheatmap (version 1.0.12). Pairwise comparisons between developmental time points were made using DEseq2, with significant genes considered as *P-*adj < 0.01 and log_2_FC ≥ 1. For the TRAP data set, highly depleted genes (TRAP/input < 0.6 in at least three out of four developmental time points) were excluded prior to enrichment analysis. We noticed samples derived from the *Ezh1* cKO cultures had a batch effect between samples originating from two independent litters and RNA sequencing runs. To account for this, we included this batch information within our DESeq2 differential model. GO enrichment analysis and gene set enrichment analysis (GSEA) were performed using clusterProfiler (version 4.2.2) (Yu et al. 2012). General processing and exploration of data were performed using the tidyverse packages (Wickham et al. 2019). Plots were prepared using ggplot2 (version 3.3.6) (Wickham 2016), ggVennDiagram (version 1.2.2) (Gao et al. 2021), and ggalluvial (version 0.12.3) (Brunson 2020).

#### Cell type marker genes

For the identification of cerebellar cell type-specific genes, we used data from a previously published scRNA-seq data set from the adult mouse brain (Saunders et al. 2018). Subclusters were combined as necessary to obtain one metacell per each major cerebellar cell type (granule cells, Purkinje cells, unipolar brush cells, stellate/basket cells, Golgi cells, astrocytes/Bergmann glia, oligodendrocytes, microglia, endothelial cells, and pericytes/mural cells). To obtain a list of marker genes for each metacell, normalized counts for each cell type were scaled by *z*-score and sorted, and cutoff values were identified using the elbow point method. For each cell type, only genes that had a *z*-score of at least 1.5× higher than the next highest were included. The identified cell type marker genes are listed in Supplemental Table S1.

### Ex vivo organotypic slices

P8 cerebella were dissected from C57BL/6J or *Ezh1*^*wt/wt*^;*Atoh1-Cre* or *Ezh1*^*fl/fl*^;*Atoh1-Cre* mouse brains in HBSS containing 2.5 mM HEPES (pH 7.4), 46 mM D-glucose, 1 mM CaCl_2_, 1 mM MgSO_4_, and 4 mM NaHCO_3_ (referred to here as HBSS with extra glucose) on ice. The dissection medium was then removed, and pCIG2-Venus or pCIG2-cMAP3-tdTomato DNA was diluted to 0.5 µg/µL in HBSS with extra glucose. The cerebella were soaked in the DNA for 15–20 min on ice and were transferred one at a time into the well of an electroporation chamber (Protech International, Inc. CUY520P5 platinum electrode L8xW5xH3 with 5-mm gap) on ice. The cerebella were electroporated dorsal to ventral for 50 msec at 80 V for a total of five pulses with an interval of 500 msec between pulses using an electro-square-porator (ECM 830, BTX Genetronics). The cerebella were then removed from the chamber, recovered in HBSS with extra glucose for 10 min on ice, and embedded in 3% agarose in HBSS containing regular glucose (30 mM D-glucose), and 250-µm coronal slices were made using a Leica VT1000S vibratome set at a speed of 3 and frequency of 6. Slices were then placed on Millicell CM 0.4-µm culture plate inserts in a six-well plate with 1.5 mL of culture medium (BME, 25 mM D-glucose, 1× glutamine, 1× ITS, 1× Pen-Strep) below the insert. For the cMAP3 experiments, slices were incubated for 6, 12, 48, and 72 h at 35°C and 5% CO_2_ before fixing with 4% PFA/4% sucrose/PBS for 2 h at room temperature. For the UNC1999 experiments, 200 nM UNC1999 in DMSO (or an equivalent volume of DMSO for the control conditions) was added to the culture medium just prior to plating the cerebellum. The organotypic slices were incubated for 60 h at 35°C and 5% CO_2_ before fixing with 4% PFA/4% sucrose/PBS for 2 h at room temperature.

### Immunostaining and imaging

#### Immunostaining

For immunostaining of organotypic slices, fixed slices were washed three times in PBS for 30 min each time at room temperature, permeabilized, and blocked overnight at 4°C in blocking solution (1× PBS, 0.3% Triton X-100, 10% normal donkey serum [NDS]). The sections for cMAP3 experiments were then incubated overnight at 4°C with rabbit anti-GFP (1:2000; Thermo Fisher Scientific A-11122, RRID:AB_221569), goat anti-tdTomato (1:200; Sicgen AB8181-200), and mouse anti-Calbindin (CALB1; 1:1000; Swant 300, RRID:AB_10000347) primary antibodies diluted in 1× PBS/0.3% Triton X-100/10% NDS. The sections for the UNC1999 and *Ezh1* cKO experiments were incubated overnight at 4°C with rabbit anti-GFP (1:2000; Thermo Fisher Scientific A-11122, RRID:AB_221569) and mouse anti-Calbindin (CALB1; 1:1000; Swant 300, RRID:AB_10000347) primary antibodies diluted in 1× PBS/0.3% Triton X-100/10% NDS. Subsequently, the sections were washed three times for 20 min each time in 1× PBS/0.3% Triton X-100/10% NDS at room temperature and incubated overnight at 4°C with secondary antibodies (Thermo Fisher Scientific A-21206, RRID:AB_2535792; Thermo Fisher Scientific A-21203, RRID:AB_141633; Thermo Fisher Scientific A-11058, RRID:AB_2534105; Abcam ab175658, RRID:AB_2687445) diluted 1:500 in 1× PBS/0.3% Triton X-100/10% NDS. The sections were then washed four times for 30 min each time, adding DAPI to the next to last wash of the slices for the UNC1999 experiments; the remaining agarose was carefully removed; and the sections were mounted with Molecular Probes ProLong Gold antifade mounting media (Invitrogen P36934).

For immunostaining of GC cultures, cells grown on 16-well chamber slides were fixed with 4% formaldehyde for 10 min, washed with PBS, permeabilized, and blocked in blocking solution for 1 h at room temperature. The cells were then incubated overnight at 4°C with the following primary antibodies: mouse anti-γH2AX (1:1000; Millipore 05-636-I, RRID:AB_2755003), rabbit anti-Caspase3 (1:500; Cell Signaling Technology 9664, RRID:AB_2070042), rabbit anti-H3K27me3 (1:1000; Cell Signaling Technology rabbit anti-H3K27me3 9733, RRID:AB_2616029), and mouse anti-NeuN (1:500; Millipore MAB377, RRID:AB_2298772). The cultures were washed with washing buffer (1× PBS, 0.3% Triton X-100, 3% NDS) three times for 10 min each time and incubated for 1 h at room temperature with secondary antibodies diluted 1:500 in washing buffer (Thermo Fisher Scientific A-21206, RRID:AB_2535792; Thermo Fisher Scientific A-21202, RRID:AB_141607; Thermo Fisher Scientific A-31572, RRID:AB_162543; and Thermo Fisher Scientific A-31570, RRID:AB_2536180).

#### Imaging

The samples were imaged with a Carl Zeiss Axiovert 200M/Perkin Elmer Ultraview spinning disk confocal microscope equipped with 25× and 63× objectives, and images were acquired and viewed using Volocity 3D image analysis software (RRID:SCR_002668). Exposure times were kept consistent within experiments.

#### Quantification

Images were exported from Volocity software, and figures were made in Adobe Photoshop and Illustrator. For the UNC1999 and *Ezh1* cKO slice culture experiments, cell number was counted manually using the “count” tool, and the distance of migration was measured manually using the “ruler” tool in Photoshop. Venus-positive cells with two processes emanating from the cell soma were considered bipolar, and those with more than two processes emanating from the soma were considered multipolar. Coimmunostaining and imaging the slices with mouse anti-Calbindin to visualize the Purkinje cell layer (PCL) and with DAPI to define the EGL and IGL aided in defining the EGL, ML, and PCL of the cerebellar cortex. The percentage of bipolar and multipolar GCs in the ML was calculated out of the total number of Venus-positive cells (*n* = 593 cells for the control condition and *n* = 880 cells for the UNC1999 condition in the UNC1999 experiment, and *n* = 378 wild-type cells and *n* = 512 *Ezh1* cKO cells in the *Ezh1* cKO experiment). Cells for which polarity could not be determined were excluded from the analysis. The distance of migration was determined by measuring the distance from the cell soma to the outermost edge of the parallel fiber track for regions of interest in lobes on the surface of the slice. For regions of interest in internal areas of a slice, where two lobes and their parallel fiber tracks were adjacent, the outermost edge of each parallel fiber track was estimated to be located half the distance between the parallel fiber tracts and Purkinje cell layers for each lobe. For the UNC1999 experiment, GC migration distance was quantified from a total of 868 cells for DMSO or 2334 cells for UNC1999, and for the *Ezh1* cKO experiment, from a total of 981 cells for wild type or 1066 cells for *Ezh1* cKO. Each replicate in the UNC1999 experiment included slices from two to three P8 mouse pups per condition. Regions of interest were imaged from five replicates for the DMSO condition and from four replicates for the UNC1999 condition. For the *Ezh1* cKO experiment, slices were prepared from three wild-type and three *Ezh1* cKO P8 mouse pups, and regions of interest were imaged from all three animals for each condition.

Quantification of immunostained GC cultures was performed using Fiji (RRID:SCR_002285). For the analysis of γH2AX-positive foci, γH2AX images were imported to Fiji, converted to binary grayscale images, and overlaid with nuclear staining (H3K27me3). The number of γH2AX foci in each nucleus was counted manually. γH2AX-positive foci were counted in a total of 409 cells from seven reaggregates for the DMSO condition and 354 cells from seven reaggregates for UNC1999. For the analysis of active Caspase3 staining, the number of active Casp3-positive cells and NeuN-positive nuclei in cultured GC reaggregates was counted manually from eight reaggregates for PBS, 20 reaggregates for DMSO, and 18 reaggregates for UNC1999. For the quantification of NeuN signal, mean fluorescence intensity was measured in reaggregate cultures, which were defined using the “freehand” tool. NeuN signal was quantified in eight reaggregates each for DMSO and UNC1999 using the “measure” tool in Fiji.

### Quantification and statistical analysis

Sample size was determined based on prior studies. No statistical methods were used to predetermine sample size. Data were excluded based on insufficient quality (assessed immediately after acquisition and before analysis). Statistical tests to determine outliers were not performed, and outliers were not excluded from the analysis. Data on bar graphs are presented as mean ± standard error of the mean. Data on box plots indicate median, first and third quartiles (lower and upper hinges), and smallest and largest observations (lower and upper whiskers, excluding outliers). Statistical analyses were performed in R using the stats and rstatix packages. Pairwise comparisons were made using unpaired Student's *t*-test. Multiple comparisons were made with analysis of variance (ANOVA) followed by Tukey HSD post hoc test or, where appropriate, with pairwise *t*-test followed by correction for multiple comparisons with the Benjamini and Hochberg (BH)/FDR method. Nonparametric data were analyzed with the Kruskal–Wallis test followed by Dunn's post hoc test. Comparisons between proportions were performed using Fisher's exact test, correcting for multiple comparisons using the FDR method. The level of statistical significance was set at *P* < 0.05.

### Data availability

ChIP-seq and RNA-seq data sets have been deposited to GEO under accession number GSE223487.

## Supplementary Material

Supplement 1

Supplement 2

Supplement 3

Supplement 4

Supplement 5
