# Supplementary material for: Histone bivalency regulates the timing of cerebellar granule cell development
Source: Genes Dev. 2023 Jul 1;37(13-14):570–89. doi: 10.1101/gad.350594.123 (PMC10499015; doi:10.1101/gad.350594.123)
Supplement: Supplement 1 [file Supplemental_Figures_S1_S8.pdf]

Supplemental Figures

Histone bivalency regulates the timing of cerebellar granule cell development

Kärt Mätlik[1], Eve-Ellen Govek[1], Matthew R. Paul[2], C. David Allis[3], Mary E. Hatten[1, #]

[1] Laboratory of Developmental Neurobiology, Rockefeller University, 10065, New York, NY, USA
[2] Bioinformatics Resource Center, Rockefeller University, 10065, New York, NY, USA
[3] Laboratory of Chromatin Biology and Epigenetics, Rockefeller University, 10065, New York, NY, USA
[#] Corresponding author: hatten@rockefeller.edu

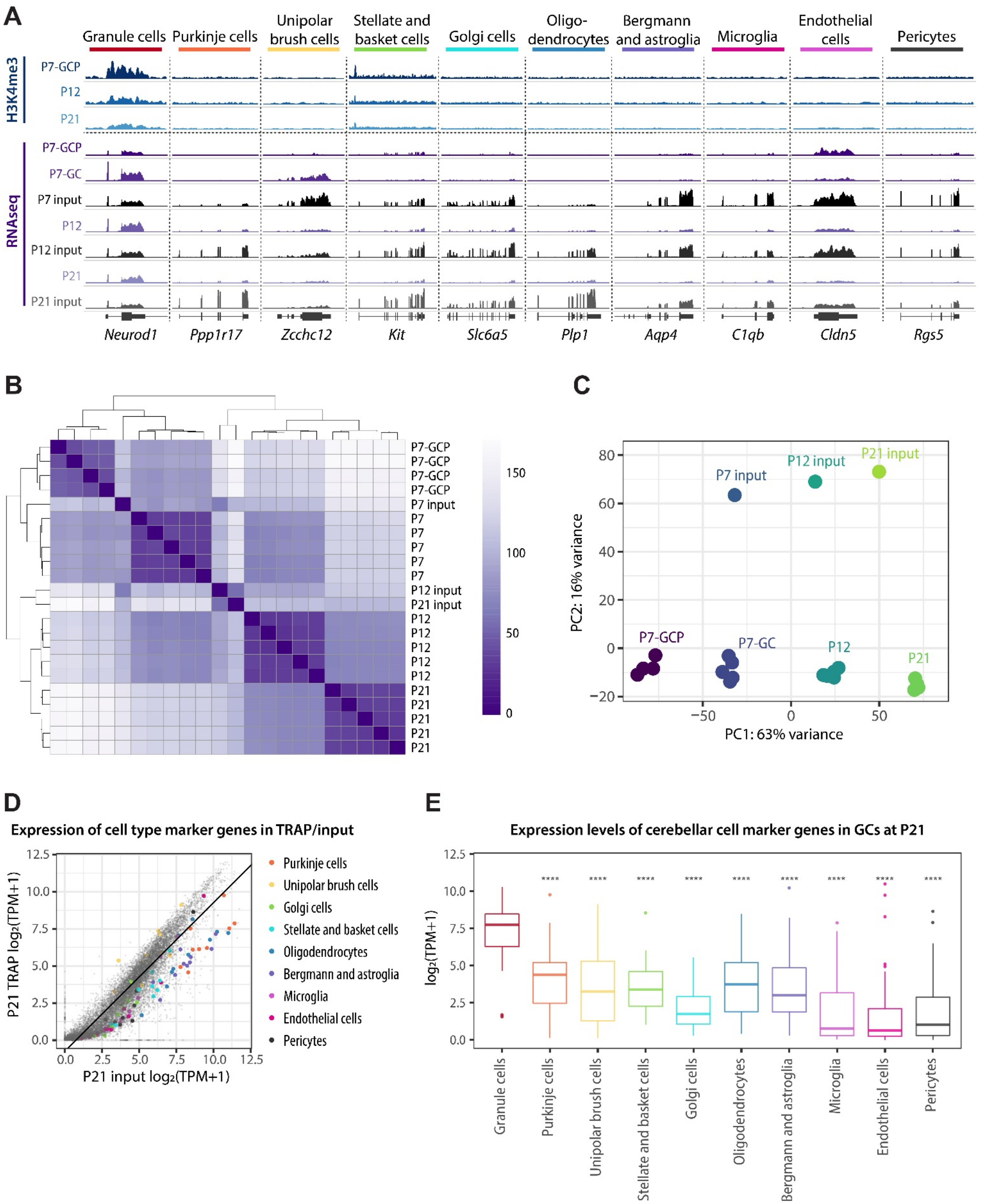

Supplemental Figure S1, related to Figure 1. Isolation of GC-specific chromatin and RNA from the postnatal cerebellum. **A** Genome browser view of representative RNA-seq and H3K4me3 ChIPseq signal at marker genes of cerebellar cell types. A representative gene is shown for each major cerebellar cell type, identified using

scRNA-seq data from the adult cerebellum (Saunders et al. 2018). Representative ChIP-seq and TRAP RNA-seq samples are shown. H3K4me3 (n=2-3 samples/group) and H3K27me3 (n=4-5 samples/group) ChIP-seq was performed on chromatin isolated from GCPs (P7) or from GC nuclei sorted using FANS (P12 and P21). RNA-seq was performed on TRAP RNA isolated from P5-P7 *Tg(Atoh1-Egfp-L10a)* GCPs (P7-GCP, n=4 samples/group), or from the cerebellar lysates of *Tg(Neurod1-Egfp-L10a)* mice at P7 (P7-GC), P12 and P21 (n=5 mice/group). For input RNA-seq, individual input samples at each age were pooled in equal amounts to yield one input per developmental time point. **B** TRAP RNA-seq sample similarity matrix. **C** Principal component analysis on TRAP RNAseq samples. **D** Scatterplot showing the expression of marker genes of cerebellar cells in P21 TRAP samples relative to input. The top 10 most highly specific genes for each cell type are shown. The line denotes the cut-off level for identifying genes depleted in TRAP samples relative to input. **E** The expression of cell type marker genes in P21 GCs. One-way ANOVA, followed by Tukey HSD *post hoc* test. Correction for multiple comparisons was performed considering all comparisons but significances are shown only for comparisons with granule cells. **** $p < 0.0001$.

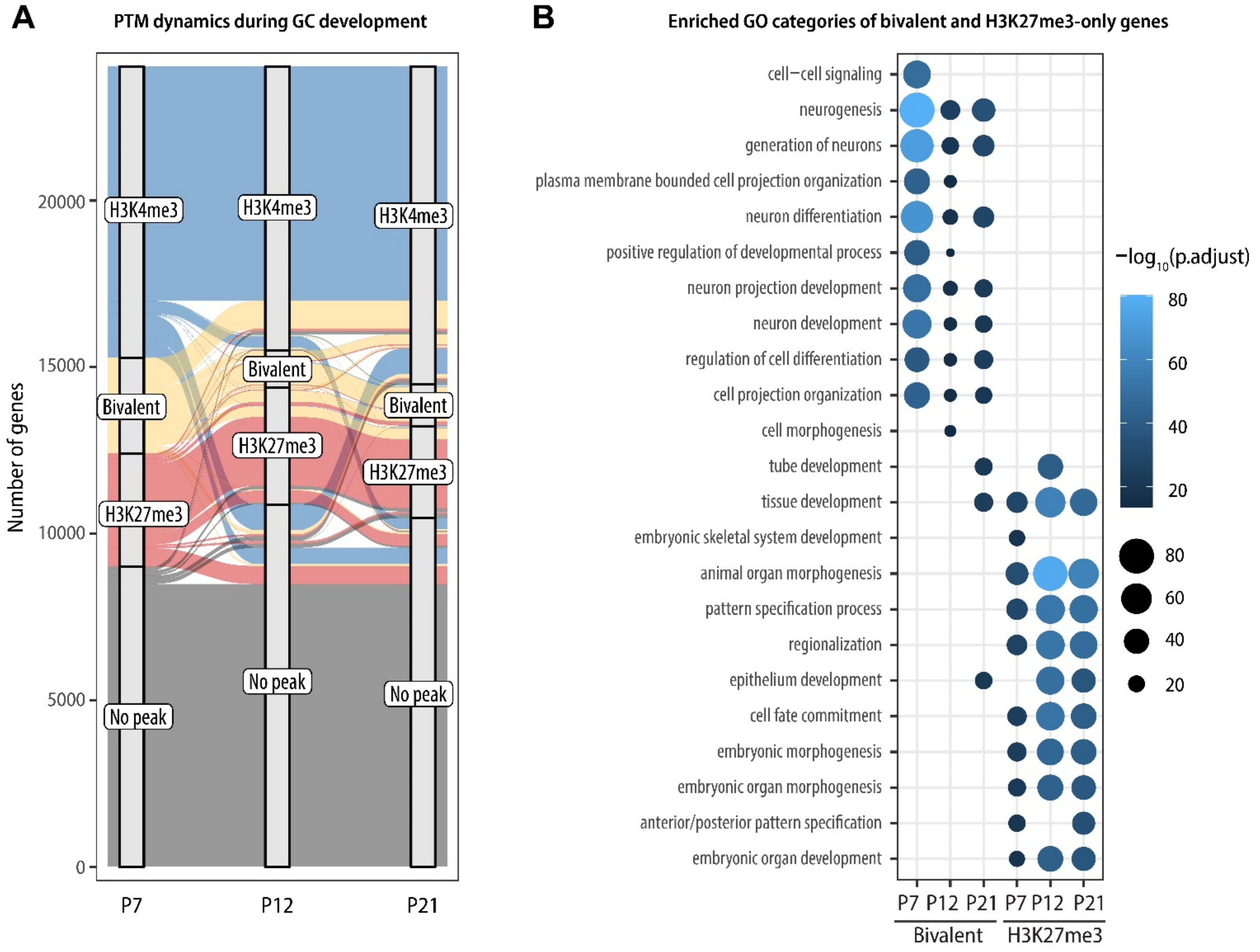

Supplemental Figure S2, related to Figures 2 and 4. **A** Alluvial plot showing the dynamics of promoter-proximal histone PTMs between P7 and P21. Groups that contain fewer than 0.5% of included genes are omitted for simplicity. The majority of H3K4me3-only and no-peak genes are stable throughout development, whereas bivalent and H3K27me3-only genes are more likely to change their PTM status. **B** Enriched GO (biological process) categories of genes with bivalent or H3K27me3-only promoters at key stages of GC development. GO categories were identified using clusterProfiler. The GO Biological Process categories were sorted by the adjusted P-value and the top 10 enriched categories are shown for each age.

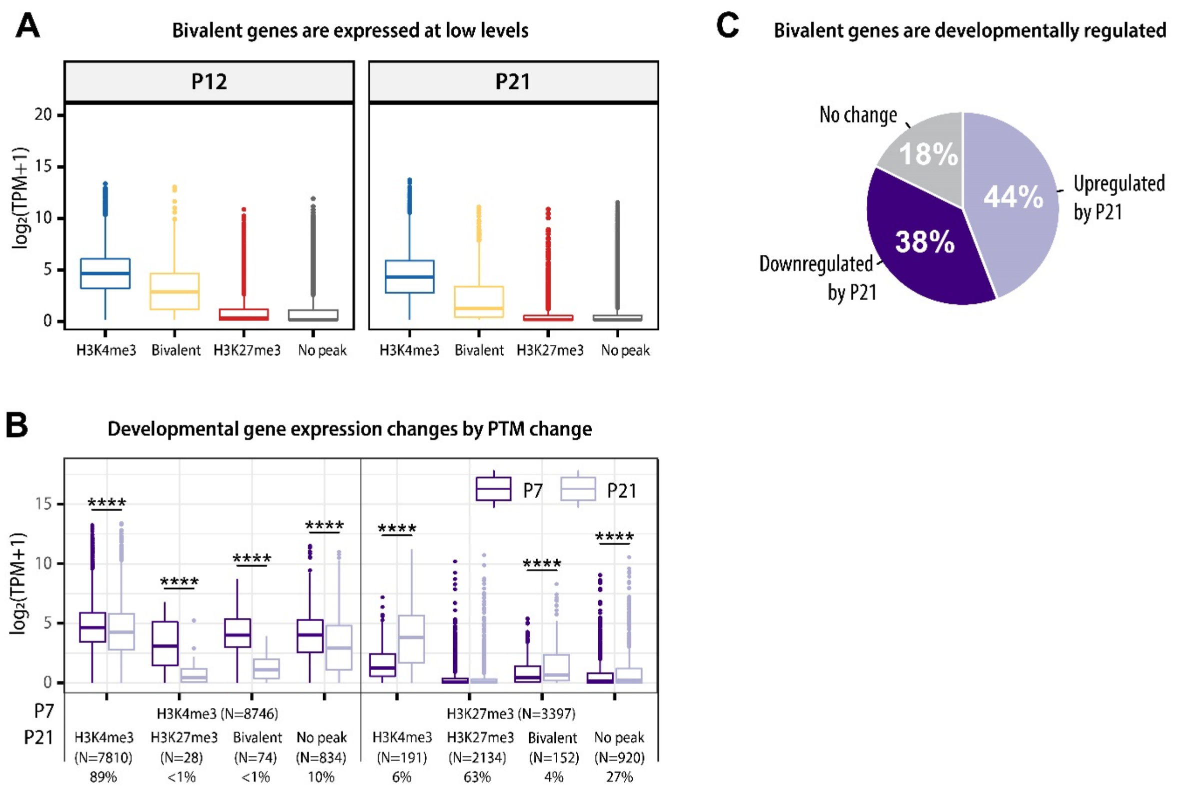

Supplemental Figure S3, related to Figure 4. Changes in bivalency correlate with gene expression. **A** The expression of H3K4me3-only, bivalent, H3K27me3-only, and no-peak genes at P12 and P21. **B** Developmental changes in PTM status at the TSS are associated with developmental gene expression changes between P7 GCPs and P21 GCs. P7 H3K4me3-only and P7 H3K27me3-only genes are shown. Pairwise t-test, adjusted for multiple comparisons using the BH method. ** $p < 0.01$, **** $p < 0.0001$. **C** Pie chart depicting the percentage of bivalent genes that are upregulated, downregulated, or do not change in expression between P7 and P21.

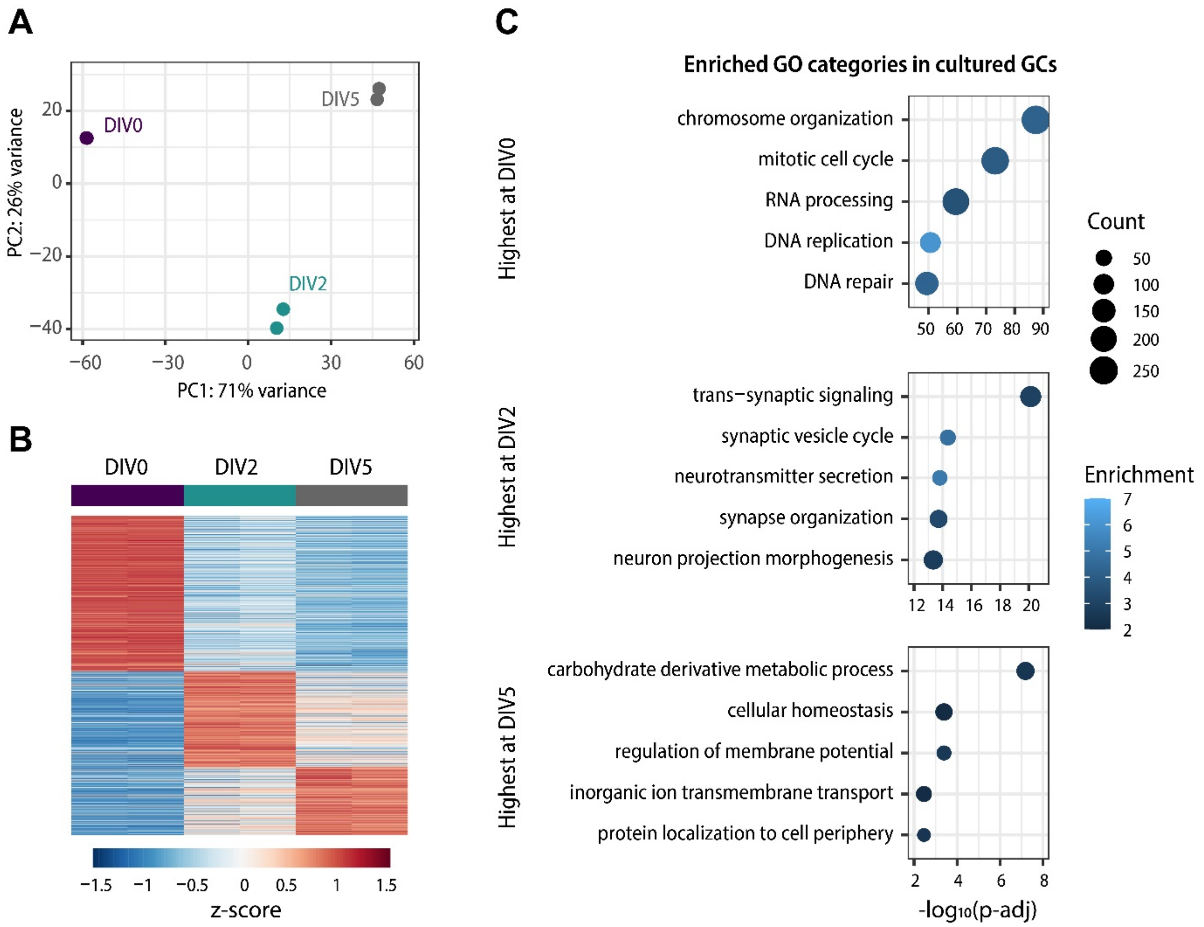

Supplemental Figure S4, related to Figure 6. Cultured GCs follow *in vivo* GC developmental trajectories. **A** Principal component analysis on RNA-seq samples isolated from cultured GCs at DIV0, DIV2 and DIV5, cultured in the presence of DMSO. **B** Heatmap depicting differentially expressed genes between cultured GCs at DIV0, DIV2, and DIV5. DE genes (p-adj < 0.05) were identified by pairwise comparisons between groups using DESeq2 and sorted by the highest expressed genes at each age. **C** Gene Ontology analysis of the highest expressed genes in each group. The GO biological process categories were sorted by the adjusted P-value and the top 5 enriched non-redundant categories are shown for each group.

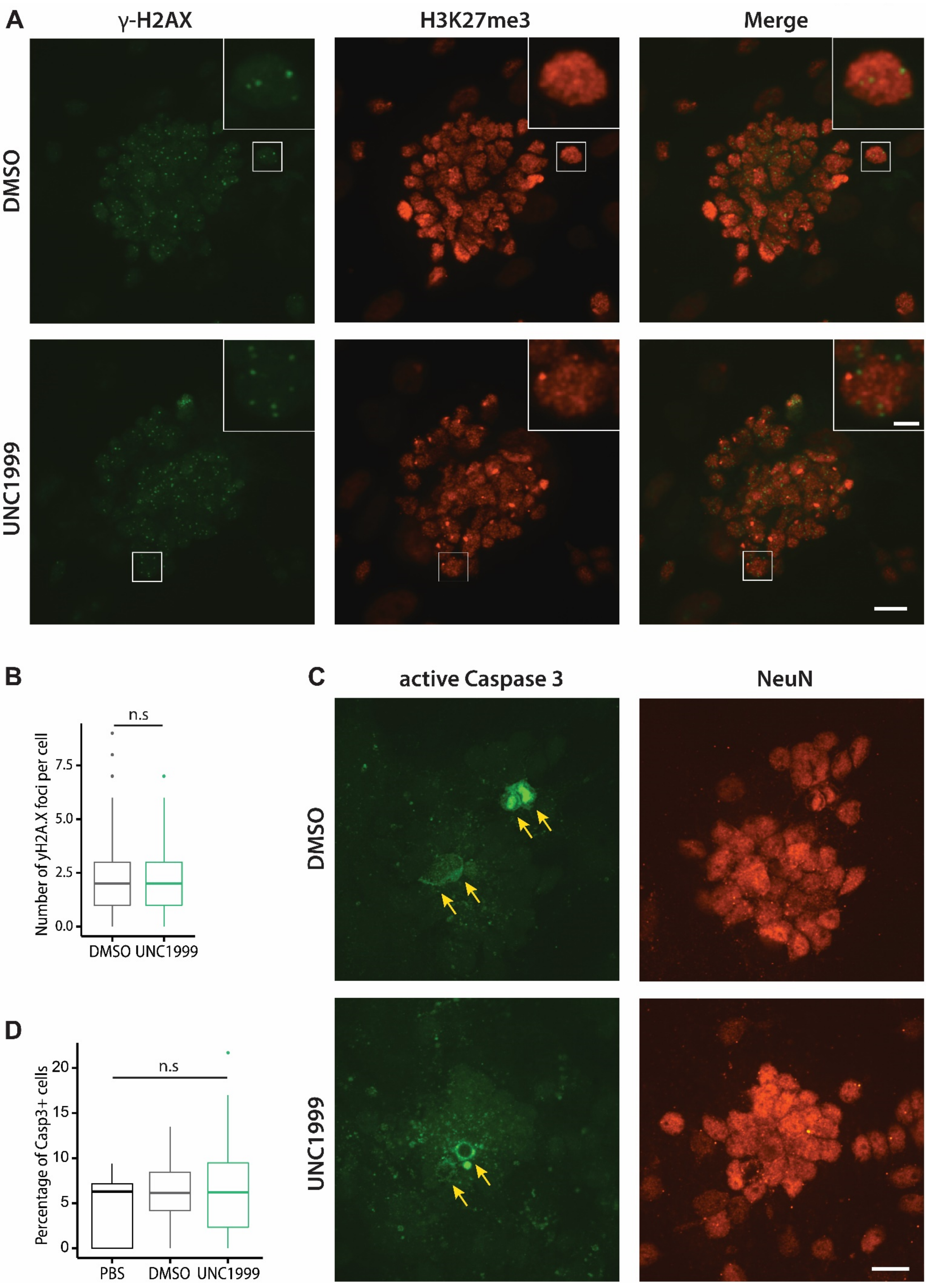
A
γ-H2AX
H3K27me3
Merge
DMSO
UNC1999
B
n.s
Number of γH2A.X foci per cell
0.0
2.5
5.0
7.5
DMSO
UNC1999
C
active Caspase 3
NeuN
DMSO
UNC1999
D
n.s
Percentage of Casp3+ cells
0
5
10
15
20
PBS
DMSO
UNC1999

Supplemental Figure S5, related to Figure 6. Analysis of DNA damage and cell death in cultured GCs after UNC1999 treatment. **A** Representative images from DIV5 GCs cultured in the presence of DMSO and UNC1999 and stained with antibodies against γ-H2AX and H3K27me3. Scale bars: 8 μm and 2 μm (inset). **B** Quantification of γ-H2AX foci per cell. γ-H2AX foci were counted from 409 cells from 7 reaggregates for the DMSO condition and 354 cells from 7 reaggregates for the UNC1999 condition. Unpaired t-test. n.s, not significant. **C** Representative images of active Caspase3 and NeuN staining of DIV5 GC cultures. Scale bar, 12 μm. **D** Quantification of the percentage of active Casp3-positive cells in reaggregates. Active Casp3-positive GCs were counted from 8 reaggregates for the PBS condition (19 active Casp3-positive cells out of 330 total), 20 reaggregates for the DMSO condition (58/843), and 18 reaggregates for the UNC1999 condition (56/804). Data points represent the percentage of active Casp3-positive cells in each reaggregate. Unpaired t-test. n.s, not significant.

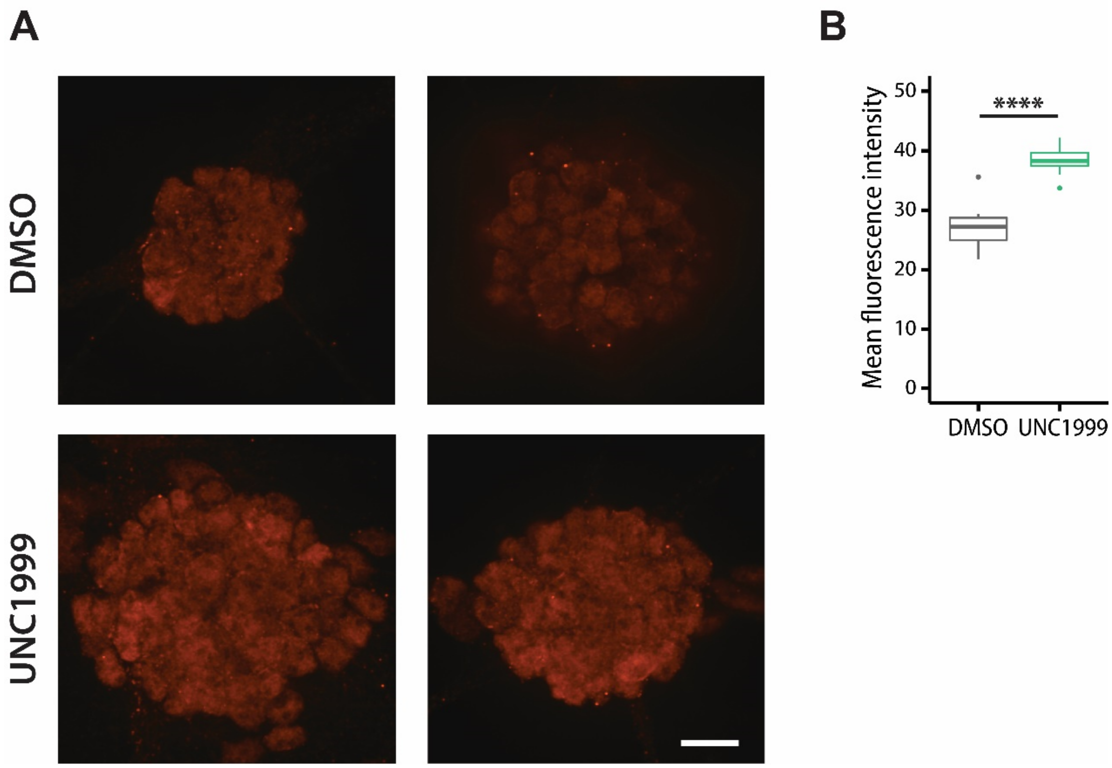

Supplemental Figure S6, related to Figure 6. NeuN expression in cultured GCs after UNC1999 treatment. **A** Representative images of DIV5 GC reaggregates stained with antibody against RBFOX3/NeuN. Reaggregate cultures support GC proliferation and the presence and increase in NeuN expression indicates those GCs that have exited the cell cycle and are undergoing maturation. Scale bar, 8 µm. **B** Quantification of mean fluorescence intensity in GC reaggregates. N = 9 reaggregates (DMSO) and 8 reaggregates (UNC1999). Unpaired t-test, **** $p < 0.0001$.

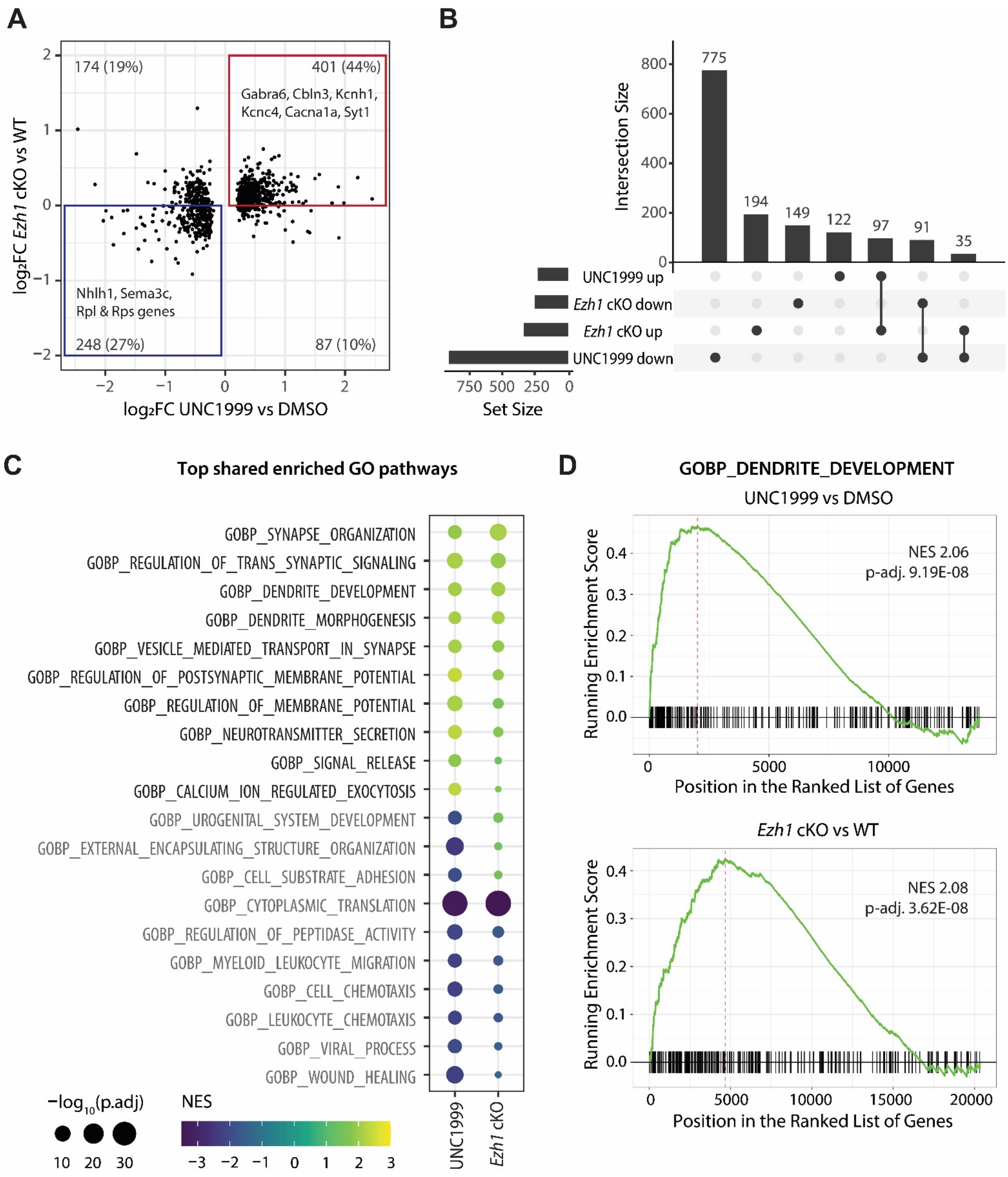

Supplemental Figure S7, related to Figures 6 and 7. Gene expression in *Ezh1* cKO GCs at DIV5. **A** Comparison of gene expression changes in GCs at DIV5 in response to UNC1999 treatment and in *Ezh1* cKO mice. Genes differentially expressed after treatment with UNC1999 are shown, and selected shared up- and downregulated genes are listed. n=2 replicate cultures in the UNC1999 experiment and n=4-5 cultures from individual mice in

the *Ezh1* cKO experiment (n=4 wild-type and n=5 *Ezh1* cKO mouse pups). **B** Upsetplot depicting shared enriched pathways identified with each treatment using GSEA. Pathways with Normalized Enrichment Score (NES) > 0 are indicated with 'up' and pathways with NES < 0 are indicated with 'down'. **C** The top 10 most significant positive and negative enriched GO biological process categories in the UNC1999 experiment that were also significantly enriched in *Ezh1* cKO GCs. Categories with a significant positive enrichment with both UNC1999 treatment and *Ezh1* cKO are indicated with black text. **D** GSEA plots showing significant positive enrichment of GO biological process 'dendrite development' after both UNC1999 treatment compared to DMSO of cultured wild-type GCs and in cultured GC prepared from *Ezh1* cKO mice compared to wild-type littermates. NES, Normalized Enrichment Score.

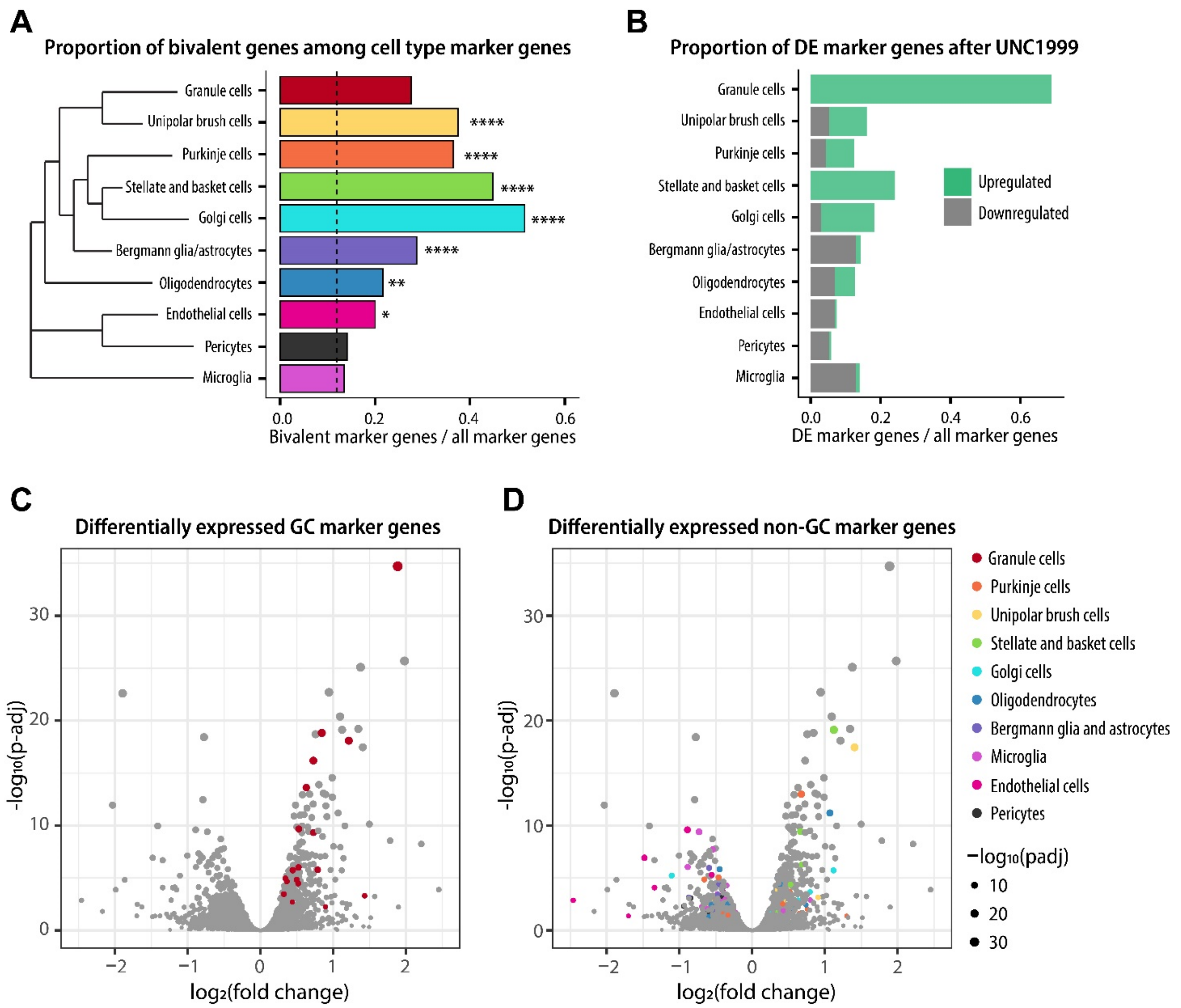

Supplemental Figure S8. Regulation of cell type marker gene expression by bivalency in developing GCs. **A** The proportion of bivalent genes among marker genes of cerebellar cells at P7. The genome-wide proportion of bivalent genes is indicated with a dashed line. Pairwise Fisher test was performed between the genome-wide proportion of bivalent genes and the proportion of bivalent genes among each cell type marker genes, using the rstatix package. P-values were adjusted for multiple comparisons using the fdr method. * $p < 0.05$, ** $p < 0.01$, *** $p < 0.001$, **** $p < 0.0001$. **B** The proportion of differentially expressed marker genes of cerebellar cells after UNC1999 treatment. **C-D** Volcano plots showing the differential expression of GC marker genes (**C**, highlighted in red) and marker genes of other cerebellar cells (**D**) in response to UNC1999 treatment.
